# Mechanistic Continuum from Stepwise to Concerted Proton-Coupled Electron Transfer Pathways at a Synthetic Tricopper Cluster

**DOI:** 10.1021/jacs.6c00324

**Published:** 2026-03-27

**Authors:** Saikat Mondal, Preston Myers, Emily N. Doss, Weiyao Zhang, Shiyu Zhang

**Affiliations:** Department of Chemistry & Biochemistry, The Ohio State University, 100 West 18th Avenue, Columbus, Ohios 43210, United States

## Abstract

Multicopper oxidases (MCOs) couple the oxidation of metal or organic substrates with the four-electron, four-proton proton-coupled electron transfer (PCET) conversion of O_2_ to H_2_O. This transformation requires at least four Cu ions: a Type I (T1) Cu site and a trinuclear Cu cluster composed of mononuclear Type II and binuclear Type III Cu centers (T2/T3). While the potential of the T2/T3 cluster remains relatively invariant (0.36–0.40 V vs NHE), that of the T1 sites varies widely among different MCOs (0.34–0.76 V vs NHE). Herein, we employ a synthetic tricopper cluster to elucidate how changes in electron-transfer driving force (Δ*G*_ET_) influence the mechanism of PCET from a Cu­(II,II,II)-oxo cluster to a Cu­(II,I,I)-hydroxo cluster, which models one of the PCET steps involved in the reductive regeneration of the Cu­(I,I,I) state in MCOs. We find that three mechanistic pathways—electron transfer–proton transfer (ET–PT), proton transfer–electron transfer (PT–ET), and concerted proton–electron transfer (CPET)—are all accessible by tuning the electron-transfer driving force and temperature. The well-defined spectroscopic signatures of ET and PT intermediates enable quantitative kinetic analysis that resolves the relative contributions of each pathway as the mechanism evolves from ET–PT to CPET to PT–ET. These results reveal a mechanistic continuum rather than a discrete switch between stepwise and concerted PCET processes.

## Graphical abstract

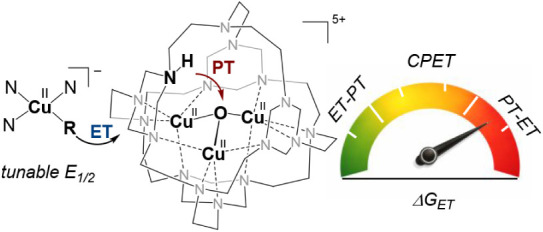

## Introduction

Multicopper oxidases (MCOs) are a class of enzymes that catalyze the conversion of dioxygen (O_2_) to water (H_2_O) while facilitating the oxidation of a wide range of metal ions and organic substrates. These enzymes have attracted attention from human health to potential applications as electrocatalysts in biofuel cells.^1–3^

Oxygen reduction reaction (ORR) at MCOs requires at least four Cu ions: a Type I (T1) Cu site and a trinuclear Cu cluster composed of a mononuclear Type II (T2) and a binuclear Type III (T3) Cu center (Figure 1A).^4–8^ The T1 site receives electrons from the metal or organic substrates and delivers them to the T2/T3 cluster, where O_2_ is activated and reduced to H_2_O.^4,5,9–11^

**Figure 1:**
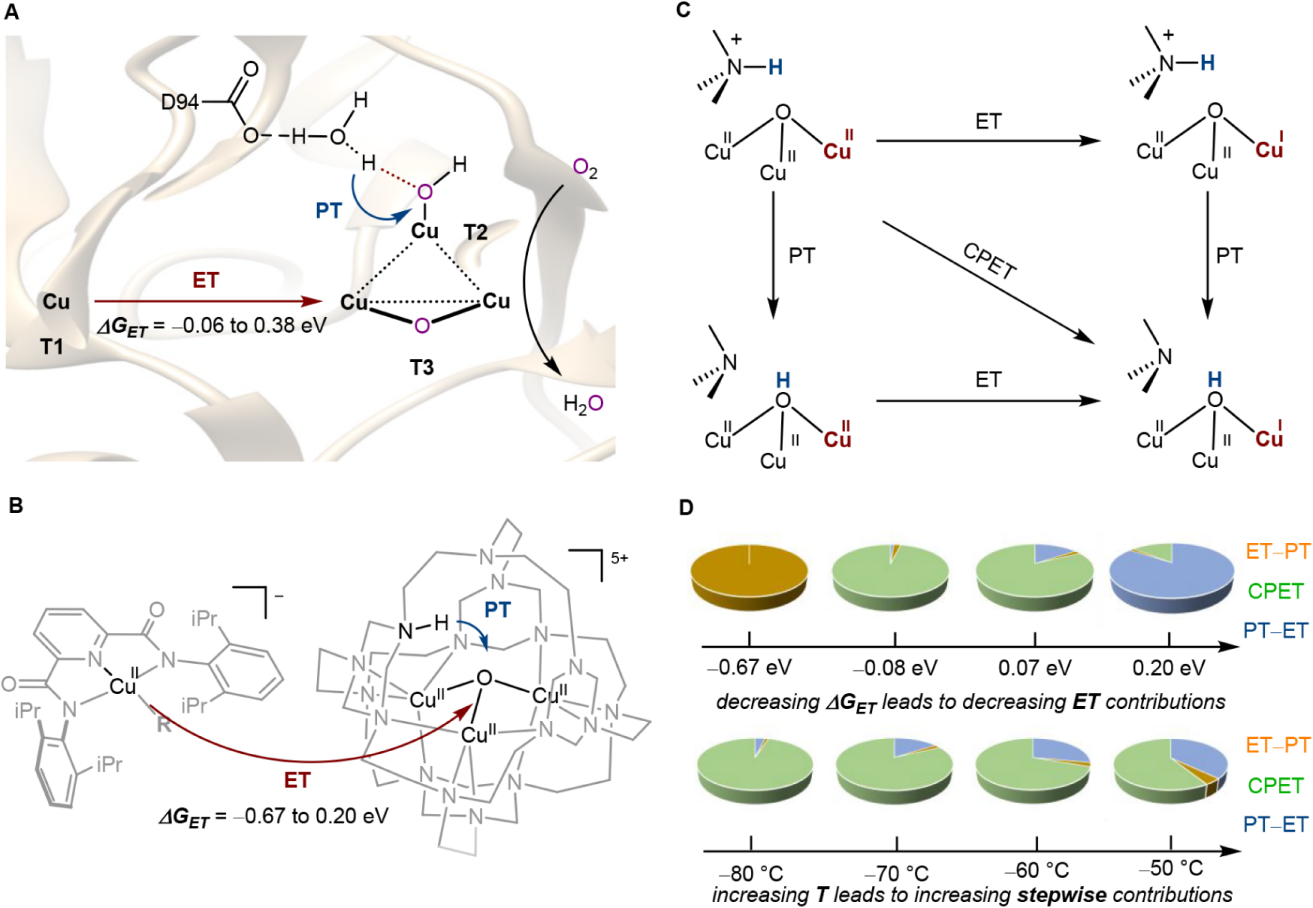
(A) Illustration of the tricopper active site in MCOs. The red arrow marks the flow of electrons (e^–^), and the blue arrow marks the flow of protons (H^+^). (B) Multiple-site PCET at a synthetic tricopper cluster investigated in this study. The red arrow indicates ET from the T1 model to the tricopper cluster, and the blue arrow indicates PT from the secondary coordination sphere to the tricopper center. (C) Mechanistic pathways for the PCET conversion of [**LH**Cu_3_(II,II,II)­(O)]^5+^ to [**L**Cu_3_(II,II,I)­(O**H**)]^5+^, showing three possible mechanisms of PCET. (D) Contribution of each mechanistic pathway to PCET as a function of the electron transfer driving forces (Δ*G*_ET_) and temperature (*T*).

Interestingly, the redox potential of the T1 site is highly variable, ranging from 0.34 V (vs NHE) in low-potential laccases to 0.78 V (vs NHE) in high-potential laccases.^12,13^ In contrast, the T2/T3 cluster exhibits relatively invariant redox potentials, typically within a narrow range of 0.36–0.40 V (vs NHE).^14^ The flexible redox potential of the T1 site, compared to the narrow and conserved range of the T2/T3 cluster, is believed to be evolutionarily advantageous: the T1 center modulates electron transfer (ET) driving force, while the trinuclear T2/T3 site provides a stable platform for efficient O_2_ reduction.^5,13^

While it is well-established that the variable redox potential of the T1 site plays a role in tuning O_2_ reduction reactivity, its influence on the mechanism of proton-coupled electron transfer (PCET) at the T2/T3 cluster remains underexplored.^5^ Studies by Hammarström, Fukuzumi, and Dempsey have shown that the mechanism of PCET can switch between concerted electron–proton transfer (CPET) and stepwise electron-transfer/proton-transfer (ET–PT or PT–ET) pathways, depending on the relative driving forces for electron and proton transfer.^15–23^ These findings raise an interesting question: Does variation in the T1 redox potential alter the PCET mechanism at the T2/T3 site similarly?^24^

Directly probing this multielectron, multiproton PCET process in native enzymes is challenging because of the overlapping spectroscopic features of the T1 and T2/T3 sites.^4–8^ This limitation motivated us to develop synthetic tricopper clusters as model systems to elucidate the fundamental PCET processes at tricopper oxo/hydroxo clusters. Previously, we synthesized a series of fully encapsulated tricopper complexes that serve as synthetic models of the T2/T3 cluster in multicopper oxidases (Figure 1B).^7–26^ Building upon this well-defined tricopper system, we herein introduced a redox-active motif outside the coordination cage to model the outer-sphere ET, analogous to the role of the T1 copper site in native MCOs (Figure 1B).

We found that variation in the redox potential of this outer-sphere ET center alters the PCET mechanism at the tricopper site (Figure 1C). At higher ET driving forces (Δ*G*_ET_ = −0.67 eV), the reaction proceeds via a stepwise ET–PT pathway, whereas at moderate driving forces (Δ*G*_ET_ = −0.08 to 0.07 eV), the mechanism shifts to CPET. Lastly, at lower ET driving forces (Δ*G*_ET_ = 0.20 eV), a PT–ET sequence becomes the dominant mechanism (Figure 1D). As the spectroscopic signatures of both ET and PT intermediates were directly observable, we quantified the relative contributions of all three pathways (CPET vs ET–PT vs PT–ET). The results revealed a continuous mechanistic transition across the PCET landscape as a function of the ET driving forces.

## Results and Discussion

### PCET Thermodynamics from [LHCu_3_(II,II,II)­(O)]^5+^ to [LCu_3_(II,II,I)­(OH)]^4^+^^

The tricopper­(II,II,II) oxo cluster was synthesized following a previously reported procedure.^25^ This species is denoted as [**LH**Cu_3_(II,II,II)­(O)]^5+^, where **LH** represents the protonated form of the cage-like ligand, in which one of the N sites in the secondary coordination sphere is protonated as ammonium (R_3_N^+^–H, Figure 1B). This protonation leads to an overall +5 charge for the complex.^25^

Reduction of [**LH**Cu_3_(II,II,II)­(O)]^5+^ with a chemical reductant affords mixed-valent species [**L**Cu_3_(II,II,I)­(O**H**)]^4+^, where **L** denotes the deprotonated form of the ligand, lacking a proton in the secondary coordination sphere. Upon reduction of the tricopper core from Cu_3_(II,II,II) to Cu_3_(II,II,I), the central oxo ligand becomes more basic and abstracts a proton from the secondary coordination sphere, yielding a μ_3_-hydroxo-bridged cluster (Figure 1C).

The coupled movement of the electron and proton in this transformation constitutes a PCET process (Figure 1C) that models the first PCET step in the reductive regeneration of the T2/T3 site in MCOs.^4–11^ Elucidating the elementary steps that govern this transformation requires disentangling how ET, PT, and CPET pathways compete as a function of the thermodynamic driving force.

We previously determined the thermodynamic parameters for both the ET and PT steps. The reduction potential (*E*°) for electron transfer and the equilibrium constant (*K*_eq_) for intramolecular proton transfer were calculated from the experimentally measured *E*_1/2_ and p*K*_a_ values of the relevant species reported earlier.^25,26^ These values are summarized in Figure 2.

**Figure 2:**
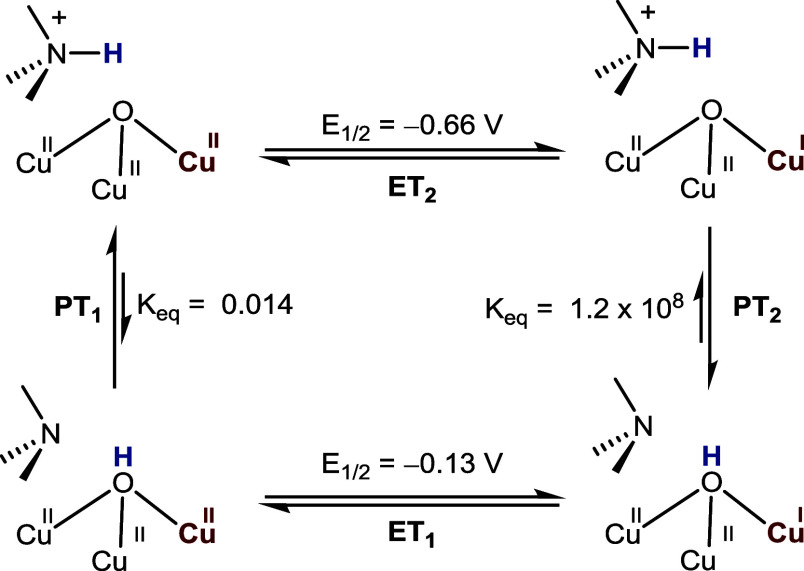
Thermodynamics of ET and PT steps from [**LH**Cu_3_(II,II,II)­(O)]^5+^ to [**L**Cu_3_(II,II,I)­(O**H**)]^4+^.

To disentangle the relative contributions of stepwise and concerted pathways, we designed a series of experiments that systematically modulate the electron-transfer driving force of the external donors. Table 1 lists the electron donors employed, spanning a Δ*G*_ET_ range from −0.67 eV to +0.20 eV vs [**LH**Cu_3_(II,II,II)­(O)]^5+^. By gradually decreasing the driving force for ET, we anticipated that the relative contributions of the three mechanistic pathways (ET–PT, CPET, and PT–ET) would vary, similar to previous studies by Hammarström, Fukuzumi, and Dempsey.^15–23^

**Table 1:** Summary of Redox Potentials, ET Driving Forces, and First-Order PCET Rate Constants Determined by UV–Vis Analysis

Reductants	*E*° vs Fc/Fc^+^ (V)^a^	Δ*G*_ET_ (eV)^b^	*k*_ET–PT_[Red](s^–1^)^c^ (Percentage)	*k*_CPET_[Red] (s^–1^)^c^ (Percentage)	*k*_PT–ET_ (s^–1^)^d^ (Percentage)
Cp_2_Co	–1.33	–0.67	(**100%**)	-	-
[PDACu^II^–Ph]^−^	–0.71	–0.08	5.62 × 10^–5^ (2.40%)	2.26 × 10^–3^ (**96.4%**)	2.83 × 10^–5^ (1.21%)
${\left[{\mathrm{PDACu}}^{\mathrm{II}}{\mathrm{-Ph}}^{{\mathrm{CF}}_{3}}\right]}^{-}$	–0.59	0.07	1.85 × 10^–6^ (1.0%)	1.54 × 10^–4^ (**83.6%**)	2.83 × 10^–5^ (15.4%)
${\left[{\mathrm{PDACu}}^{\mathrm{II}}{\mathrm{-Ph}}^{{\left({\mathrm{CF}}_{3}\right)}_{2}}\right]}^{-}$	–0.46	0.20	9.66 × 10^–7^ (2.85%)	4.58 × 10^–6^ (13.5%)	2.83 × 10^–5^ (**83.6%**)

a Redox potential measured vs Fc/Fc^+^ in acetone.

b Driving force $\Delta G_{\mathrm{ET}}\;(\mathrm{eV})=-F(E_{{\left[\mathrm{LH}{\mathrm{Cu}}_{3}(\mathrm{II},\mathrm{II},\mathrm{II})(O)\right]}^{5+}}^{{}^{\circ}}-E_{\mathrm{reductant}}^{{}^{\circ}})$ .

c *k*_ET–PT_ and *k*_CPET_ are second-order rate constants (M^–1^ s^–1^) determined as averages over three independent trials (see Supporting Information, Tables S2–S6), which are expressed as pseudo-first-order rates by multiplying by the 2 mM concentration of the excess reductant.

d *k*_PT–ET_ is the first-order rate constant determined experimentally as averages over three independent trials (see Supporting Information, Table S1).

Three mechanistic pathways are considered in analyzing the PCET process of [**LH**Cu_3_(II,II,II)­(O)]^5+^ (Figure 2). The overall rate constant (*k*_obs_) reflects the sum of contributions from the three accessible pathways—ET–PT, PT–ET, and CPET (eq 1):

1 $$k_{obs}=k_{PT-ET}+k_{ET-PT}+k_{CPET}$$

In the PT–ET pathway (Figure 2), intramolecular proton transfer (PT_1_) from the secondary coordination sphere generates a hydroxo intermediate [**L**Cu_3_(II,II,II)­(O**H**)]^5+^, which is subsequently reduced by outer-sphere ET (ET_1_). The ET–PT pathway (Figure 2) involves an initial outer-sphere ET (ET_2_) to yield [**LH**Cu_3_(II,II,I)­(O)]^4+^, followed by intramolecular PT (PT_2_) from the secondary coordination sphere R_3_N^+^–H group. Finally, in the CPET pathway (Figure 2), electron and proton transfer occurs simultaneously through a single transition state to directly generate [**L**Cu_3_(II,II,I)­(O**H**)]^4+^ from the oxo precursor. As both the ET_2_ intermediate ([**LH**Cu_3_(II,II,I)­(O)]^4+^) and the PT_1_ intermediate ([**L**Cu_3_(II,II,II)­(OH)]^4+^, Figure 2) have been previously characterized spectroscopically,^25,26^ we can experimentally differentiate these three pathways using UV–vis spectroscopy.

The stepwise ET–PT and PT–ET pathways can be further classified into PT/ET-limiting or pre-equilibrium limits with the following rate expression (see Supporting Information):^24^

2 $$k_{PT-ET}=k_{PT1},\left(PT_{1}\;\mathrm{step}\;\mathrm{is}\;\mathrm{rate-limiting}\right)$$

3 $$k_{ET-PT}=k_{ET2},\left(ET_{2}\;\mathrm{step}\;\mathrm{is}\;\mathrm{rate-limiting}\right)$$

4 $$k_{ET-PT}=K_{ET}\times k_{PT2},\left(\mathrm{pre-equilibrium}\right)$$

5 $$k_{PT-ET}=K_{PT}\times k_{ET1},\left(\mathrm{pre-equilibrium}\right)$$

Under the scenarios described in eqs 2 and 3, where the initial PT or ET step is rate-limiting, the corresponding intermediates are not observable. In contrast, if these steps occur under pre-equilibrium conditions (eqs 4 and 5), the PT or ET intermediates may be detected, provided that the experimental time scale is faster than their subsequent conversion.

### ET–PT Mechanism at High ET Driving Force

Previously, we showed that the treatment of [**LH**Cu_3_(II,II,II)­(O)]^5+^ with strong reductant cobaltocene (Cp_2_Co) in acetone at −90 °C affords a metastable species with a UV–vis band at 630 nm (ε = 3150 M^–1^ cm^–1^), which was assigned as [**LH**Cu_3_(II,II,I)­(O)]^4+^.^25^ Subsequent warming of the solution to −80 °C leads to conversion of [**LH**Cu_3_(II,II,I)­(O)]^4+^ to [**L**Cu_3_(II,II,I)­(O**H**)]^4+^ due to intramolecular proton transfer, with Δ*H* ^‡^ = 9.9 kcal mol^–1^ and Δ*S* ^‡^ = −20 cal mol^–1^ K^–1^ (Figure 3A and 3B). The negative activation entropy likely reflects the requirement to access a more ordered transition-state geometry involving a short O···N donor–acceptor distance.^27,28^ This two-step reaction sequence with a fast ET step followed by a slower PT step is diagnostic of an ET–PT (pre-equilibrium) mechanism. Therefore, our observation of ET intermediate [**LH**Cu_3_(II,II,I)­(O)]^4+^ suggests that the PCET proceeds through an ET–PT pathway at high ET driving forces (Δ*G*_ET_ < −0.67 eV, Table 1).

**Figure 3:**
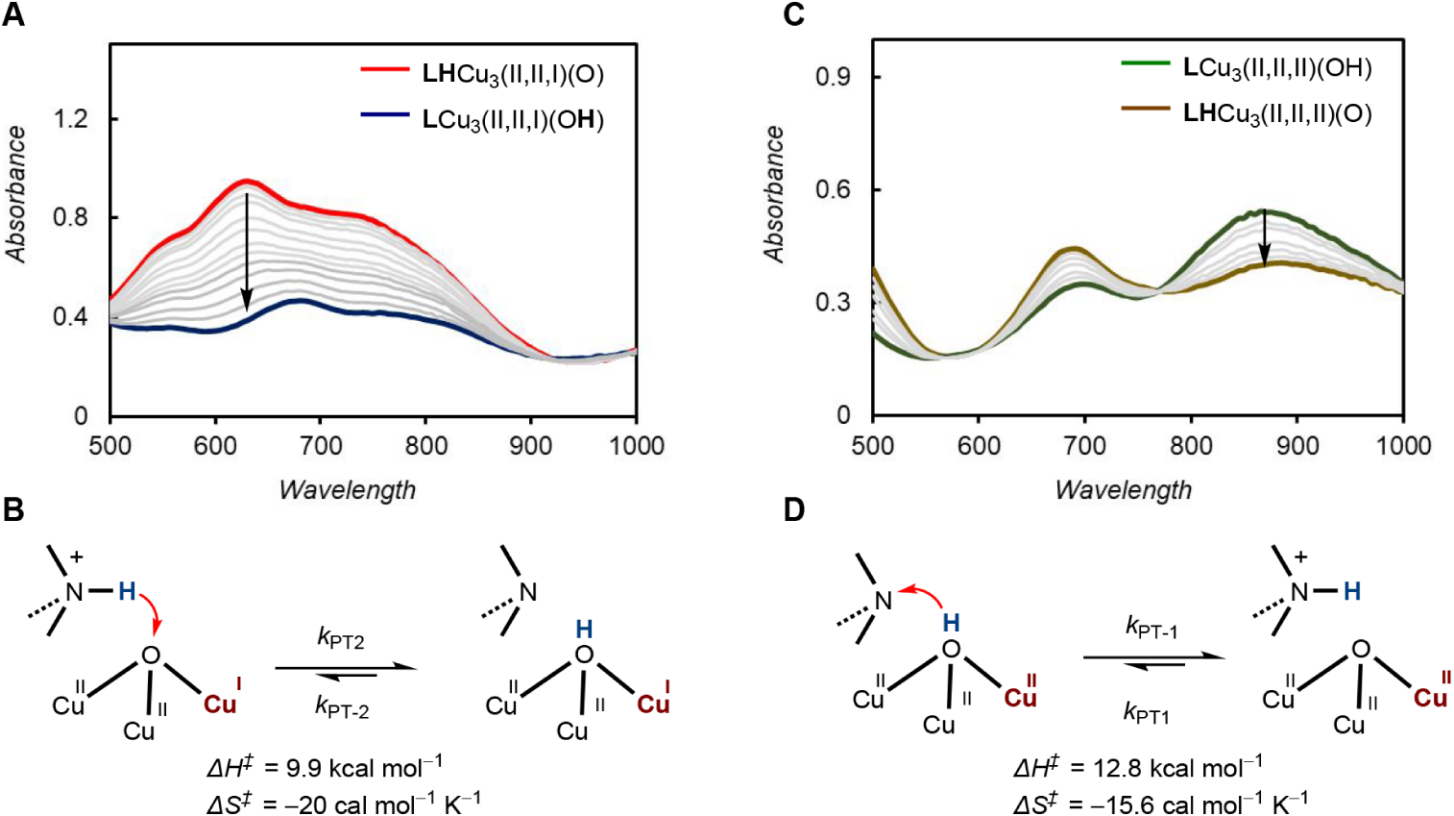
(A) UV–vis spectra of the reaction of **LH**Cu_3_(II,II,II)­(O)­(PF_6_)_5_ with one equivalent of Cp_2_Co at −80 °C (red trace), followed by intramolecular proton transfer to generate [**L**Cu_3_(II,II,I)­(O**H**)]^4+^ (blue trace). (B) Mechanism of the intramolecular proton transfer, converting [**LH**Cu_3_(II,II,I)­(O)]^4+^ to [**L**Cu_3_(II,II,I)­(O**H**)]^4+^, along with Eyring parameters for the PT_2_ step. (C) UV–vis spectra of the reaction of [**L**Cu_3_(II,II,I)­(O**H**)]^4+^ with one equivalent of Magic Blue in acetone at −90 °C (green trace), followed by intramolecular proton transfer to form [**L**Cu_3_(II,II,II)­(O**H**)]^5+^ (yellow trace). (D) Mechanism of the intramolecular proton transfer, converting [**L**Cu_3_(II,II,II)­(O**H**)]^5+^ to [**LH**Cu_3_(II,II,I)­(O)]^5+^, along with Eyring parameters for the PT_1_ step.

### Crossover from ET–PT to CPET at Moderate Δ*G*_ET_

Having established the ET–PT pathway at high ET driving force, we next investigated the PCET of [**LH**Cu_3_(II,II,II)­(O)]^5+^ at a moderate driving force with [PDACu^II^–Ph]^−^ (Δ*G*_ET_ = −0.08 eV) as the electron donor under pseudo-first-order conditions. [PDACu^II^–Ph]^−^ was prepared according to a literature procedure (see Supporting Information).^29^ With a milder reductant, no accumulation of the ET intermediate [**LH**Cu_3_(II,II,I)­(O)]^4+^ was observed at low temperature using UV–vis spectroscopy (see Supporting Information, Figure S11). Instead, the [**LH**Cu_3_(II,II,II)­(O)]^5+^ complex was cleanly converted to the [**L**Cu_3_(II,I,I)­(O**H**)]^3+^—two-electron reduction product, as [PDACu^II^–Ph]^−^ is strong enough to reduce the PCET product [**L**Cu_3_(II,II,I)­(O**H**)]^3+^ again. This observation suggests that the ET–PT (pre-equilibrium) mechanism becomes inaccessible and is instead replaced by other PCET pathways, such as ET–PT (ET-limiting), CPET, or PT–ET pathways.

To quantify the contribution of the sequential PT–ET mechanism, we first needed to measure the rate of intramolecular PT_1_ from [**LH**Cu_3_(II,II,II)­(O)]^5+^ to [**L**Cu_3_(II,II,II)­(O**H**)]^5+^ (*k*_PT1_). Although this proton transfer step is thermodynamically uphill,^26^ we can still calculate *k*_PT1_ using the equilibrium constant *K*_eq_(PT_1_) and the reverse proton transfer rate (*k*_–PT1_) using eq 6:

6 $$K_{eq}=k_{PT1}/k_{-PT1}$$

The value of *k*_–PT1_ (backward proton transfer rate) can be determined by monitoring the conversion of in situ-generated [**L**Cu_3_(II,II,II)­(O**H**)]^5+^ to [**LH**Cu_3_(II,II,II)­(O)]^5+^.

Accordingly, a solution of [**L**Cu_3_(II,II,II)­(O**H**)]^5+^ is generated by oxidation of [**L**Cu_3_(II,II,I)­(O**H**)]^4+^ with one equivalent of tris­(4-bromophenyl)­ammoniumyl hexafluorophosphate (Magic Blue) (0.67 V vs Fc/Fc^+^, Figure 3C and 3D) in acetone at −90 °C. Warming the solution to −75 °C leads to the intramolecular PT_1_, the rate of which can be measured using UV–vis spectroscopy. Under experimental conditions described above, *k*_–PT1_ values were determined at temperatures ranging from −75 to −60 °C. Combining the values of *K*_eq_ and *k*_–PT1_ in eq 2 allows us to determine the value of *k*_PT1_ as well as the barrier of activation for intramolecular PT_1_ (Δ*H* ^‡^ = 12.8 kcal mol^–1^, Δ*S* ^‡^ = −15.6 cal mol^–1^ K^–1^, see Supporting Information, Figure S21). The experimentally determined PT_1_ rates can be used as the overall rate of the stepwise PT–ET pathway since the rate of the electron transfer (ET_1_) step is expected to be much faster than the PT_1_ step in a PT-limited PT–ET scenario.

Notably, at −70 °C with [PDACu^II^–Ph]^−^ as the reductant, the observed overall PCET rate (ca. 2.34 × 10^–3^ s^–1^) is ca. two orders of magnitude faster than the contribution of the PT–ET pathway alone (ca. 2.83 × 10^–5^ s^–1^). This observation suggests that the PT–ET mechanism alone cannot account for the observed kinetics, implying that alternative pathways, such as concerted PCET or ET–PT (ET-limiting), are also simultaneously operative.

To establish a quantitative framework for these mechanistic assignments, global kinetic modeling was performed using *KinTek* Explorer.^30,31^ In the kinetic model, we incorporated parallel PT–ET, ET–PT, and CPET pathways, followed by a subsequent one-electron reduction step leading to the two-electron reduced product, [**L**Cu_3_(II,I,I)­(O**H**)]^3+^ (Scheme S1, see Supporting Information). The experimentally measured intramolecular proton-transfer rate constants (PT_1_ and PT_2_) were fixed and set to the experimental values during the fitting process,^25^ while bimolecular ET and CPET parameters were optimized to reproduce the observed kinetic traces.

The resulting fits provide a quantitative depiction of the PCET energy landscape (Table 1), in which multiple concerted and stepwise pathways coexist. Under these conditions (−70 °C, Δ*G*_ET_ = −0.07 eV), the reaction proceeds predominantly through a CPET mechanism (96.4%, Figure 4, green) with minimal contribution from stepwise PT–ET (Figure 4, blue) or ET–PT pathways (Figure 4, orange).

**Figure 4:**
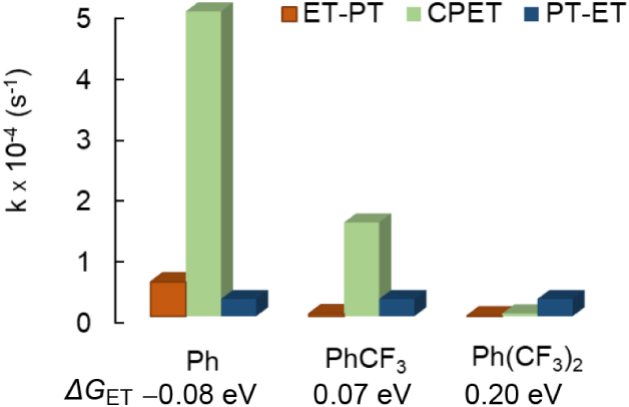
Contribution of each mechanistic pathway (ET–PT: yellow, CPET: green, PT–ET: blue) to PCET as a function of electron transfer driving forces.

### Crossover from CPET to PT–ET at Low Δ*G*_ET_

Having established CPET as the predominant pathway at Δ*G*_ET_ = −0.08 eV, we next examined how a further decrease of electron-transfer driving force modulates the mechanism of PCET using ${\left[{\mathrm{PDACu}}^{\mathrm{II}}{\mathrm{-Ph}}^{{\mathrm{CF}}_{3}}\right]}^{-}$ (Δ*G*_ET_ = 0.07 eV) and ${\left[{\mathrm{PDACu}}^{\mathrm{II}}{\mathrm{-Ph}}^{{\left({\mathrm{CF}}_{3}\right)}_{2}}\right]}^{-}$ (Δ*G*_ET_ = 0.20 eV).

In both cases, treatment of [**LH**Cu_3_(II,II,II)­(O)]^5+^ with ten equivalents of reductant showed no detectable formation of the ET intermediate [**LH**Cu_3_(II,II,I)­(O)]^4+^ by low-temperature UV–vis spectroscopy (see Supporting Information, Figures S13 and S16). Under pseudo-first-order conditions with excess reductant, clean conversion to the PCET product was observed. For the ${\mathrm{Ph}}^{{\mathrm{CF}}_{3}}$ derivative (Δ*G*_ET_ = 0.07 eV), kinetic modeling reveals a mechanistic crossover region, where CPET remains the primary pathway (83.6%), with approximately 15.4% from the PT–ET route (Table 1). This mixed regime arises due to the decreasing CPET rate as the ET step becomes less favorable (Figure 4).

Upon further reduction of ET driving force with the (CF_3_)_2_-substituted analogue (Δ*G*_ET_ = 0.20 eV), the reaction undergoes a crossover to the PT–ET mechanism (83.6%) with a substantially lower CPET contribution (13.5%). The systematic evolution from pure CPET to mixed and ultimately PT–ET behavior highlights how variations in driving force modulate the balance between electron and proton transfer events, representing a continuous mechanistic continuum within the same tricopper framework.

### Temperature-Dependent PCET Mechanism Switch

Having established how decreasing Δ*G*_ET_ reshapes the relative contributions of CPET and PT–ET pathways at a fixed temperature, we next examined whether temperature provides an additional lever to modulate the PCET mechanism. The ${\left[{\mathrm{PDACu}}^{\mathrm{II}}{\mathrm{-Ph}}^{{\mathrm{CF}}_{3}}\right]}^{-}$ (Δ*G*_ET_ = 0.07 eV) system is suitable for this study because CPET and PT–ET exhibit similar rate constants, placing the reaction near a mechanistic crossover regime. Under these conditions, changes in the activation free energies could shift the balance between the two pathways. Because proton and electron transfer barriers respond differently to temperature, we anticipated that changing temperature could alter the distribution of reaction pathways independently of Δ*G*_ET_.

To investigate the effect of temperature, we examined the reduction of [**LH**Cu_3_(II,II,II)­(O)]^5+^ by ${\left[{\mathrm{PDACu}}^{\mathrm{II}}{\mathrm{-Ph}}^{{\mathrm{CF}}_{3}}\right]}^{-}$ (Δ*G*_ET_ = 0.07 eV) over the temperature range of −80 to −50 °C (see Supporting Information, Figures S12–S15). At −80 °C, the reaction proceeds almost exclusively through the CPET pathway with a 95.3% contribution (Table 2, Figure 5). As the temperature is raised, the overall PCET rate increases, with a measurable increase in the contribution from the stepwise PT–ET pathway (3.55% to 37.5%).

**Table 2:** First-Order PCET Rate Constants (s^–1^) with ${\left[\mathrm{PDACu}{\mathrm{Ph}}^{{\mathrm{CF}}_{3}}\right]}^{-}$ as the Reductant at Different Temperatures

Temperature	*k*_ET‑PT_[Red] (s^–1^)^a^	*k*_CPET_[Red] (s^–1^)^a^	*k*_PT‑ET_ (s^–1^)^b^
–80 °C	5.42 × 10^–6^	1.78 × 10^–6^	1.46 × 10^–4^
	(1.16%)	(**95.3%**)	(3.55%)
–70 °C	2.83 × 10^–5^	3.87 × 10^–6^	1.52 × 10^–4^
	(2.10%)	(**82.5%**)	(15.4%)
–60 °C	1.40 × 10^–4^	1.21 × 10^–5^	3.43 × 10^–4^
	(2.45%)	(**69.3%**)	(28.2%)
–50 °C	5.00 × 10^–4^	5.31 × 10^–5^	7.79 × 10^–4^
	(3.99%)	(**58.5%**)	(37.5%)

a *k*_ET‑PT_ and *k*_CPE_T are second-order rate constants (M^–1^ s^–1^) determined as averages over three independent trials (see Supporting information, Tables S2–S6), which are expressed as pseudo-first-order rates by multiplying by the 2 mM concentration of the excess reductant.

b *k*_PT‑ET_ is the first-order rate constant determined experimentally as averages over three independent trials (see Supporting information, Table S1).

**Figure 5:**
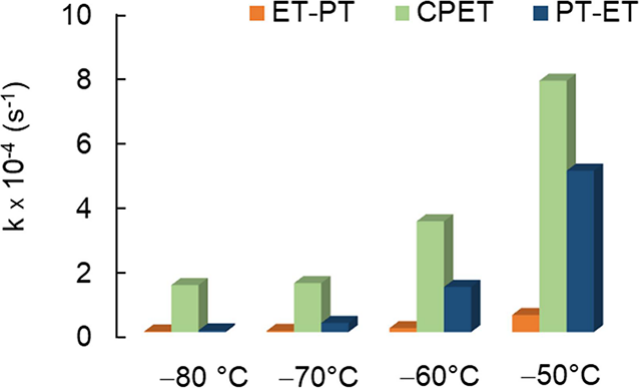
Contribution of each mechanistic pathway (ET–PT: yellow, CPET: green, PT–ET: blue) to PCET as a function of temperature with the ${\left[{\mathrm{PDACu}}^{\mathrm{II}}{\mathrm{-Ph}}^{{\mathrm{CF}}_{3}}\right]}^{-}$ derivative.

We attribute this temperature-dependent mechanism crossover to the weak temperature dependence of the CPET rate^32–34^ and the strong temperature dependence of the PT rate (Δ*H* ^‡^ = 12.8 kcal mol^–1^, Δ*S* ^‡^ = −15.6 cal mol^–1^ K^–1^). This variable-temperature study further confirms the coexistence of multiple PCET mechanisms, suggesting a mechanistic continuum rather than a discrete switch between pathways.

## Conclusions

In this work, we demonstrate that the synthetic tricopper complex [**LH**Cu_3_(II,II,II)­(O)]^5+^ provides a platform to dissect the mechanistic landscape of PCET that is relevant to multicopper oxidases. By systematically varying the outer-sphere electron transfer driving force, we observe that the PCET from [**LH**Cu_3_(II,II,II)­(O)]^5+^ to [**L**Cu_3_(II,I,I)­(O**H**)]^3+^ does not follow a single pathway but rather traverses a continuum across stepwise ET–PT, CPET, and PT–ET sequences. For a strong ET driving force, the PCET process favors a stepwise ET–PT path; at moderate driving force, a concerted PCET path dominates; and eventually, at weak ET driving force, the system shifts toward PT–ET. Variable-temperature studies further confirmed the coexistence of CPET and stepwise ET–PT or PT–ET mechanisms. At low temperatures, CPET contributes more to the overall reaction, whereas at higher temperatures, the stepwise PT–ET pathway is more important.

The ability to modulate the PCET pathway at tricopper centers by changing the outer-sphere redox potential has implications for the biological function of the T1 to T2/T3 electron transfer in MCOs. In the context of biological O_2_ reduction, the redox potential of the T1 sites in MCOs is also highly variable, serving as a redox gate that enables reductive regeneration of the fully reduced Cu_3_(I,I,I) state. Our work demonstrates that subtle differences in electron-transfer driving forces can lead to distinct PCET intermediates, a phenomenon that may also occur in low- vs high-potential laccases. These different PCET pathways and intermediates would influence catalytic overpotentials, turnover rates, and selectivity for the four-electron O_2_ to H_2_O conversion.

## Supplementary Material



## Notes

Experimental details, including characterization data, spectra, computational procedures, and results (PDF)
