# Supplementary material for: Mechanistic Continuum from Stepwise to Concerted Proton-Coupled Electron Transfer Pathways at a Synthetic Tricopper Cluster
Source: J Am Chem Soc. 2026 Mar 27;148(13):14170–6. doi: 10.1021/jacs.6c00324 (PMC13067264; doi:10.1021/jacs.6c00324)
Supplement: Supplementary file 1 [file ja6c00324_si_001.pdf]

Electronic Supporting Information for:

Mechanistic Continuum from Stepwise to Concerted Proton-Coupled Electron Transfer Pathways at a Synthetic Tricopper Cluster

Saikat Mondal, Preston Myers, Emily N. Doss, Weiyao Zhang, and Shiyu Zhang*

Department of Chemistry and Biochemistry, The Ohio State University, 100 West 18th Avenue, Columbus, Ohio 43210, United States of America

*Correspondence to: zhang.8941@osu.edu

Contents

1.Materials and Methods S2
2.Synthesis and Characterization of [TBA]PDACu^{II}-Ph S2
3.Synthesis and Characterization of [TBA]PDACu^{II}-PhCF$_3$ S4
4.Synthesis and Characterization of [TBA]PDACu^{II}-Ph(CF$_3$)$_2$ S6
5.EPR details S7
6. Derivation of Rate law S9
7. UV-Vis spectroscopy and Kinetic Simulation S11
Kinetic study of the reaction between [**LH**Cu$_3$(II,II,II)(O)](PF$_6$)$_5$ and PDACu^{II}Ph S11
Kinetic study of the reaction between [**LH**Cu$_3$(II,II,II)(O)](PF$_6$)$_5$ and PDACu^{II}PhCF$_3$ S12
Kinetic study of the reaction between [**LH**Cu$_3$(II,II,II)(O)](PF$_6$)$_5$ and PDACu^{II}Ph(CF$_3$)$_2$. S14
Kinetic study of the reaction between [**LH**Cu$_3$(II,II,II)(O)](PF$_6$)$_5$ and Cr(C$_6$H$_6$)$_2$ S15
Kinetic study of the reaction between [**LH**Cu$_3$(II,II,II)(O)](PF$_6$)$_5$ and Me$_{10}$Fc........ S16
Kinetic study of the reaction between [**L**Cu$_3$(II,II,I)(OH)](PF$_6$)$_4$ and Magic Blue S16
Kinetic modelling and simulation parameters S18
7. Reference S29

1. Materials and Methods

General Experimental details: All syntheses and experiments were performed under a nitrogen atmosphere in an MBraun glovebox or using standard Schlenk techniques unless otherwise noted. Dichloromethane, tetrahydrofuran, acetonitrile, fluorobenzene, pentane, and diethyl ether were dried and degassed under nitrogen using a Pure Process Technologies (PPT, Nashua, NH) solvent purification system and stored over 4 Å molecular sieves. Elemental analysis was performed by Midwest Micro Lab (Indianapolis, IN, http://midwestlab.com/). [N,N'-bis(2,6-diisopropylphenyl)-2,6-pyridinedicarboxamido] acetonitrilecopper(II) (PDACu^{II}(MeCN)) and [TBA][PDACu^{II}F] were synthesized as previously described.[1] Tris(4-bromophenyl)ammoniumyl hexafluorophosphate (magic blue),[1] [**L**Cu^{II}Cu^{I}Cu^{I}(OH)](PF$_6$)$_3$,[2] [**L**Cu^{II}Cu^{II}Cu^{I}(OH)](PF$_6$)$_4$,[2] [**LH**Cu^{II}Cu^{II}Cu^{II}(O)](PF$_6$)$_5$,[3] were prepared according to the published methods. Diphenylzinc, bis(4-trifluoromethylphenyl)zinc and di(3,5-bis(trifluoromethyl)phenyl)zinc were synthesized according to previous literature.[4]
All reagents were obtained from standard suppliers and used without further purification. NMR spectra were recorded on a Bruker Avance NEO 400 MHz instrument or a Bruker Avance III HD 600 MHz instrument and referenced to residual solvent peaks. UV-vis spectra were collected on an Agilent Cary 60 spectrophotometer outfitted with a Unisoku Unispeks cryostat (−100 °C to + 100 °C). EPR spectra were recorded on a Bruker EMXPlus X-band EPR spectrometer equipped with Coldedge variable temperature cryostat at 30 K. Cyclic voltammograms were recorded under a nitrogen atmosphere with a Biologic SP-150 potentiostat using a three-electrode system comprised of a glassy carbon working electrode, a platinum wire counter electrode, and a Ag/AgNO$_3$ (0.01 M) reference electrode. All cyclic voltammograms were recorded at room temperature with a scan rate of 100 mV/s and referenced internally to Fc/Fc+. Compounds were dissolved in an electrolyte solution consisting of 0.100 M electrochemical grade tetrabutylammonium hexafluorophosphate.

2. Synthesis and characterization of [TBA]PDACu^{II}-Ph

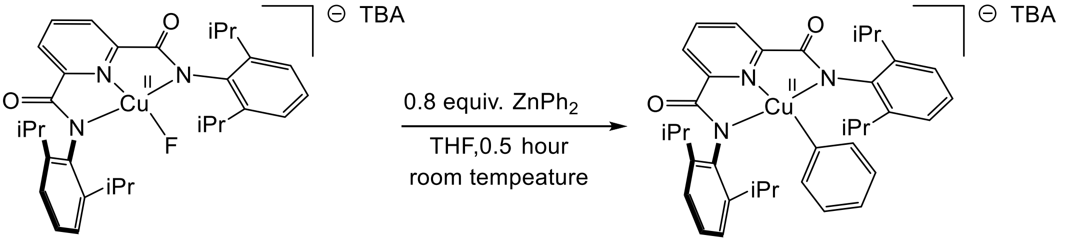

The title compound was prepared according to a literature report.[5] A stirring solution of [TBA][PDACuF] (132 mg, 0.1632 mmol) in THF (4 mL) was treated with a THF solution (4 mL) of diphenylzinc (ZnPh$_2$, 28.7 mg, 0.1307 mmol, 0.8 eq) at room temperature. The dark blue solution was stirred for 30 minutes, during which it turned to a dark red color. The resulting solution was filtered over Celite and dried in vacuo to yield a red oily solid. The crude solid was dissolved in minimal THF (~ 3 mL), after which excess Et$_2$O was added (~ 15 mL) to precipitate out unreacted [TBA][PDACu^{II}-F]. The Et$_2$O layer was decanted and dried down to yield a dark purple solid (117.4 mg, 72% yield). The product was recrystallized in THF/pentane

at −40 °C to yield dark purple crystals of [TBA]PDACu^{II}-Ph. Elemental analysis, Calcd for [TBA]PDACu^{II}-Ph, $C_{53}H_{78}Cu_1N_4O_2(C_4H_8O)_2(H_2O)_2$: C, 69.98; H, 9.43; N, 5.35. found C, 69.52; H, 9.02; N, 5.66. UV-vis (490 nm, 439 $M^{-1}cm^{-1}$).

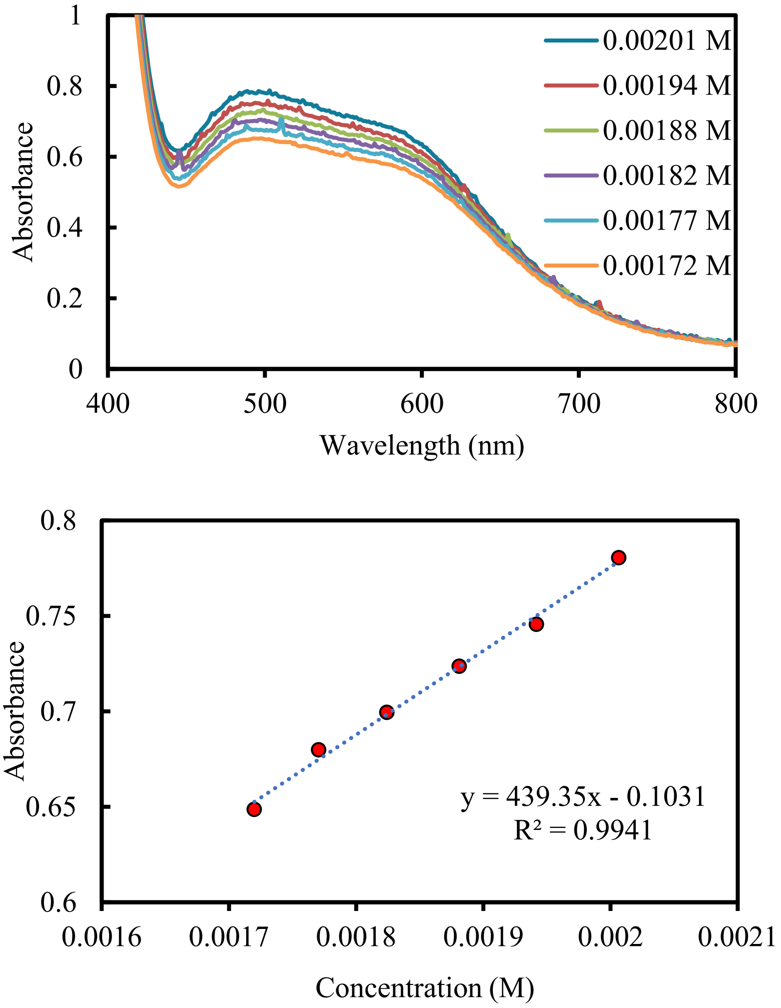

Figure S1. (A) UV-vis spectra of [TBA]PDACu^{II}-Ph in acetone at different concentrations. (B) Beer's law plot of [TBA]PDACu^{II}-Ph at λ_{max} = 490 nm gives ε = 439 $M^{-1}cm^{-1}$.

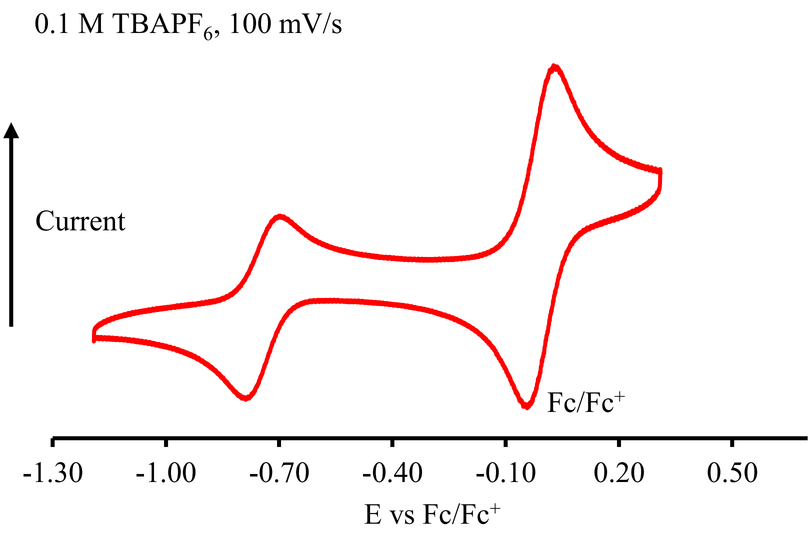

Figure S2. CV of [TBA]PDACu^{II}-Ph in 0.1 M $TBAPF_6$ acetone electrolyte at 0.1 V/s scan rate with ferrocene as internal standard.

3. Synthesis and characterization of [TBA]PDACu^{II}-$PhCF_3$

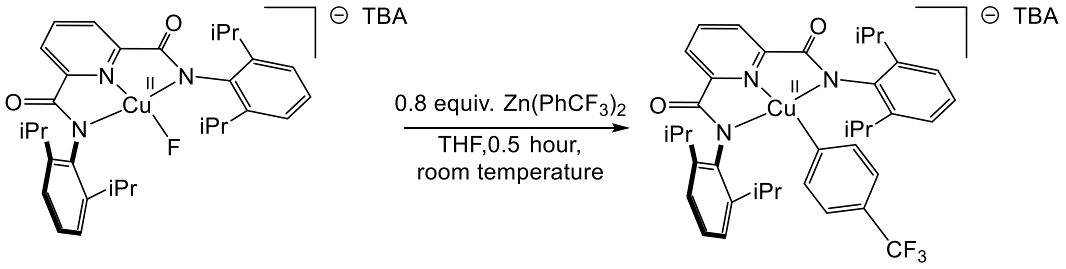

The title compound was prepared by adaptation of a literature report.[5] A stirring solution of [TBA]PDACuF (48.1 mg, 0.0595 mmol) in THF (3 mL) was treated with a THF solution (3 mL) of bis(4-trifluoromethylphenyl)zinc (16.9 mg, 0.0475 mmol, 0.8 eq) at room temperature. The dark blue solution was stirred for 30 minutes, during which it turned to a dark red color. The resulting solution was filtered over Celite and dried in vacuo to yield a red oily solid. The crude solid was dissolved in minimal THF (~ 3 mL), after which excess Et_2O was added (~ 15 mL) to crash out unreacted [TBA][PDACu^{II}-F]. The Et_2O layer was decanted and dried down to yield a purple solid (41.6 mg, 75% yield). The product was recrystallized in THF/pentane at −40 °C to yield purple crystals of [TBA]PDACu^{II}-$PhCF_3$. Elemental analysis, Calcd for [TBA]PDACu^{II}-$PhCF_3$, $C_{54}H_{77}Cu_1N_4O_2F_3$: C, 69.38; H, 8.30; N, 5.99. found C, 69.34; H, 8.31; N, 6.06. UV-vis (515 nm, 426 $M^{-1}cm^{-1}$).

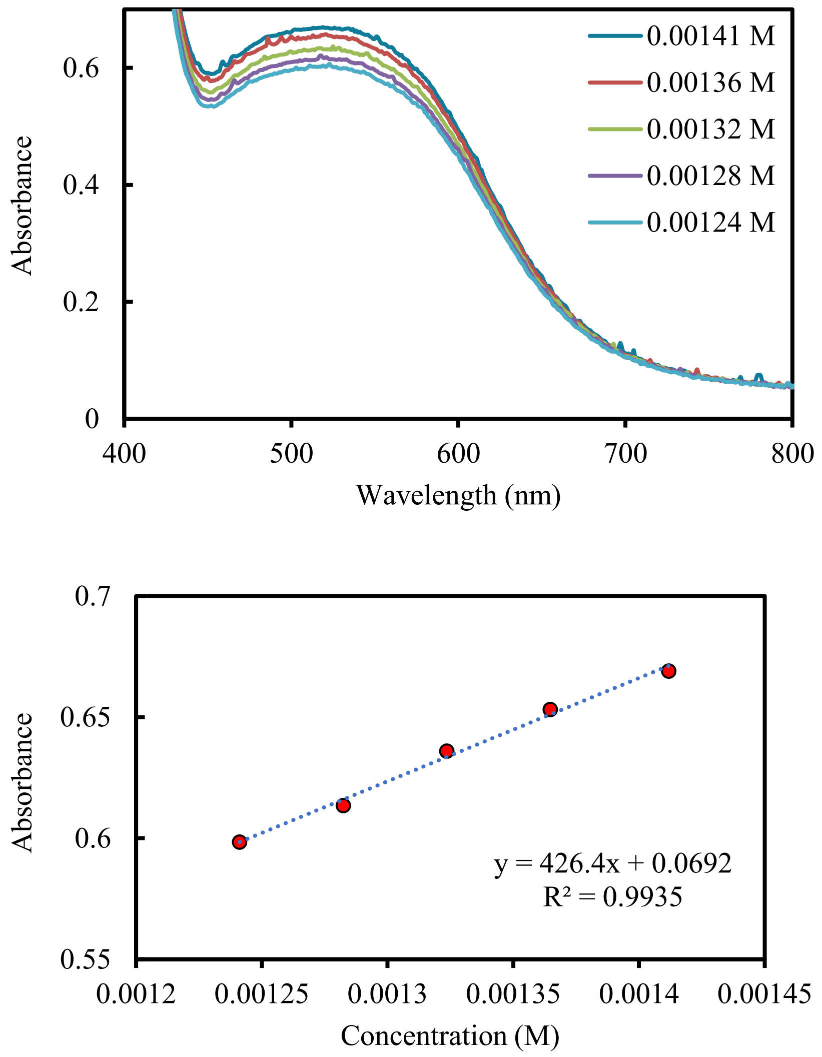

Figure S3. (A) UV-vis spectra of [TBA]PDACu^{II}-PhCF$_3$ in acetone at different concentrations. (B) Beer's law plot of [TBA]PDACu^{II}-PhCF$_3$ at λ_{max} = 515 nm gives ε = 426 M^{-1}cm^{-1}.

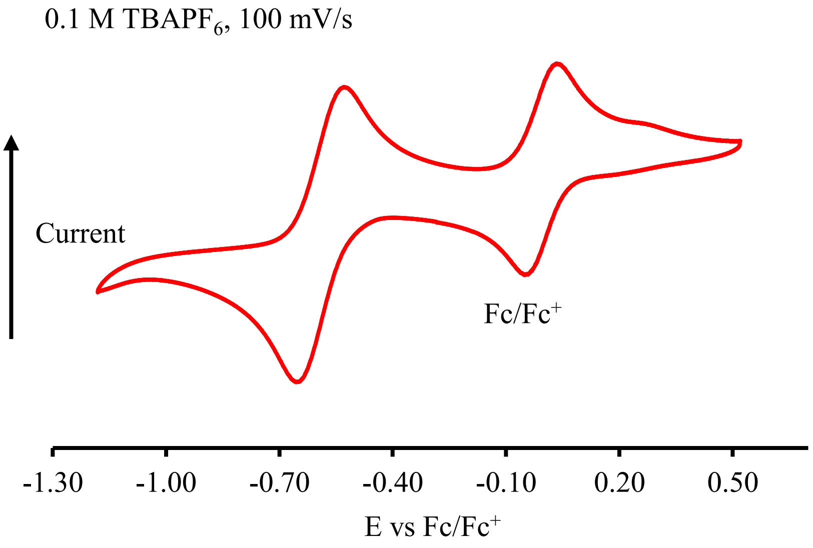

Figure S4. CV of [TBA]PDACu^{II}-PhCF$_3$ in 0.1 M TBAPF$_6$ acetone electrolyte at 0.1 V/s scan rate with ferrocene as internal standard.

4. Synthesis and characterization of [TBA]PDACu^{II}-Ph(CF$_3$)$_2$

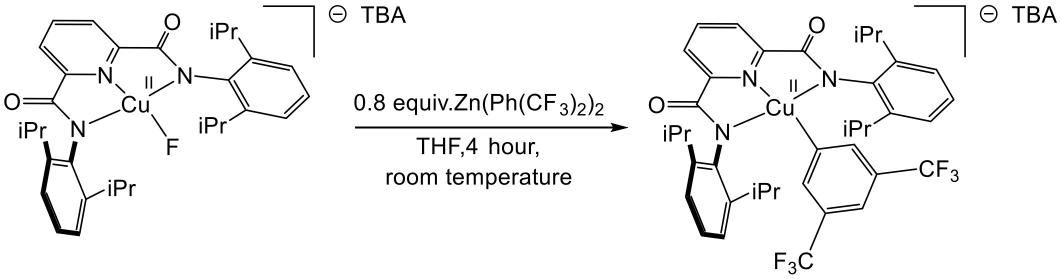

The title compound was prepared by adaptation of a literature report.[5] A stirring solution of [TBA]PDACuF (24.8 mg, 0.0307 mmol) in THF (3 mL) was treated with a THF solution (3 mL) of di(3,5-bis(trifluoromethyl)phenyl)zinc (12.1 mg, 0.0246 mmol, 0.6 eq) at room temperature. The dark blue solution immediately turned into a dark green solution. After stirring for 4 hours, the solution turned into a dark red-purple color. The resulting solution was filtered over Celite and dried in vacuo to yield a reddish-purple oily solid. The crude solid was dissolved in minimal THF (~3 mL), after which excess Et$_2$O was added (~15 mL) to precipitate out unreacted [TBA][PDACu^{II}-F]. The Et$_2$O layer was decanted and dried down to yield a purple solid (30.6 mg, 88% yield). The product was recrystallized in THF/pentane at −40 °C to yield reddish-purple crystals of [TBA]PDACu^{II}-Ph(CF$_3$)$_2$. Elemental analysis, Calcd for [TBA]PDACu^{II}-Ph(CF$_3$)$_2$, $C_{55}H_{76}Cu_1N_4O_2F_6$ (H_2O): C, 64.72; H, 7.70; N, 5.49. found C, 64.64; H, 7.67; N, 5.54. UV-vis (540 nm, 419 $M^{-1}cm^{-1}$).

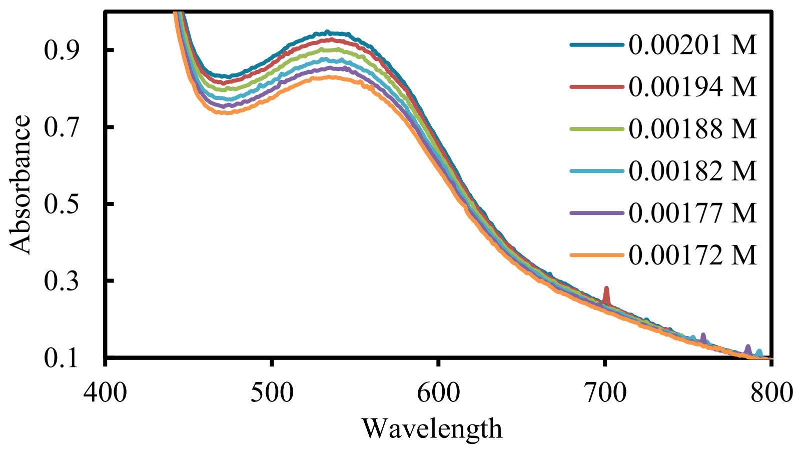

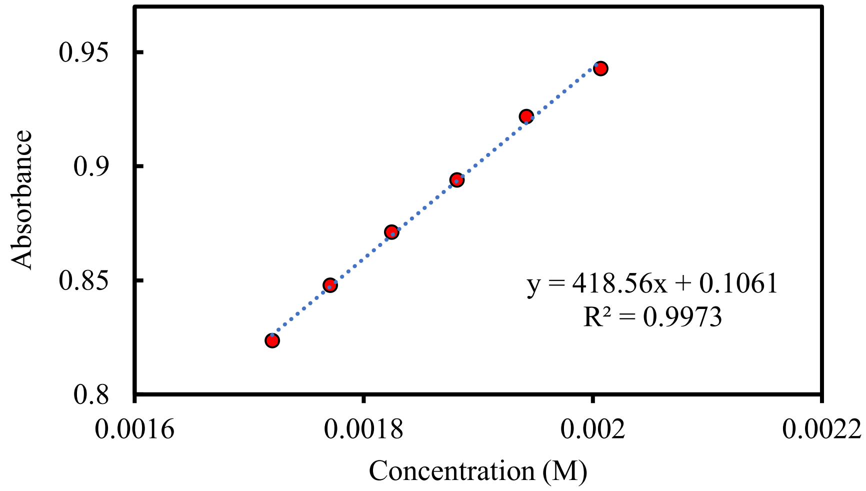

Figure S5. UV-vis spectra of [TBA]PDACu^{II}-Ph(CF_3)$_2$ in acetone at different concentrations. (B) Beer's law plot of [TBA]PDACu^{II}-Ph(CF_3)$_2$ at λ_{max} = 540 nm gives ε = 419 $M^{-1}cm^{-1}$.

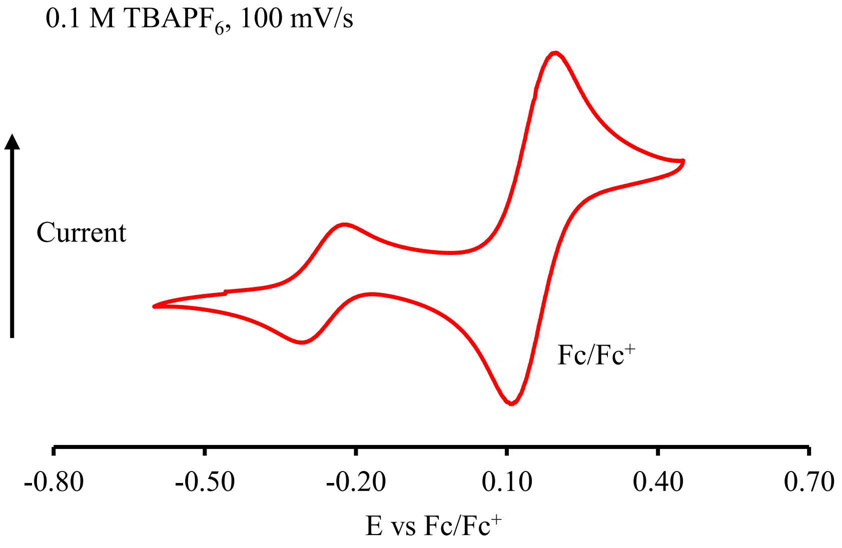

Figure S6. CV of [TBA]PDACu^{II}-Ph(CF_3)$_2$ in 0.1 M $TBAPF_6$ acetone electrolyte at 0.1 V/s scan rate with ferrocene as internal standard.

5. X-band EPR details

EPR spectra were recorded on a Bruker EMXPlus X-band EPR spectrometer equipped with Coldedge cryostat with small-volume power saturation. All samples were measured in 4mm septum-capped EPR quartz tubes (Wilmad Lab glass, 727-SQ-250MM). The spectra were collected at 5 K, unless otherwise noted, with a modulation frequency of 100 kHz, and a time constant of 40.96 ms were employed. All spectra were baseline-corrected using Igor Pro (Wavemetrics, Lake Oswego, OR) software. Spectral simulations were performed using the EasySpin toolbox with MATLAB.[6]

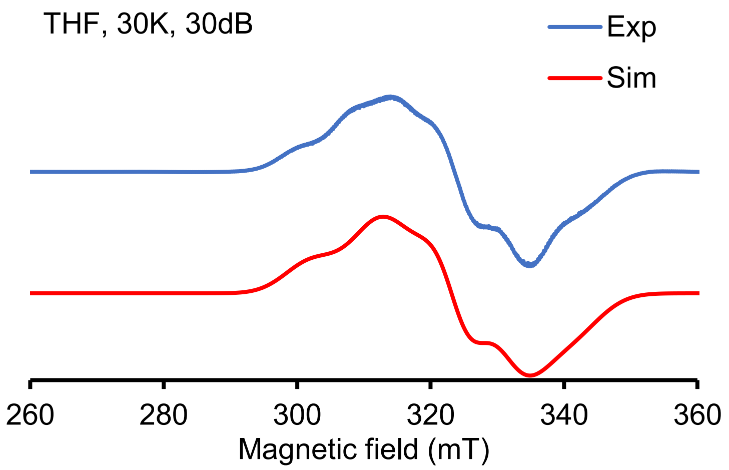

Figure S7. (A) X-band EPR spectrum of [TBA]PDACu^{II}-Ph (frozen THF, 1 mM, 30 K), g_1 = 2.09, g_2 = 2.05, g_3 = 2.135, A_1= 5 MHz, A_2= 235 MHz, A_3= 290 MHz. The experimental spectrum is shown in blue, and the simulation is depicted in red.

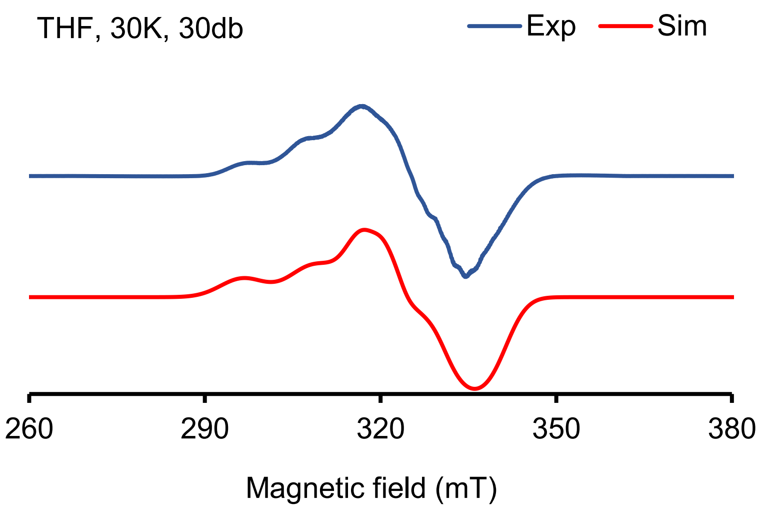

Figure S8. (A) X-band EPR spectrum of [TBA]PDACu^{II}-PhCF$_3$ (frozen THF, 1 mM), g_1 = 2.105 g_2 = 2.025, g_3 = 2.155, A_1= 70 MHz, A_2= 90 MHz, A_3= 350 MHz. The experimental spectrum is shown in blue, and the simulation is depicted in red.

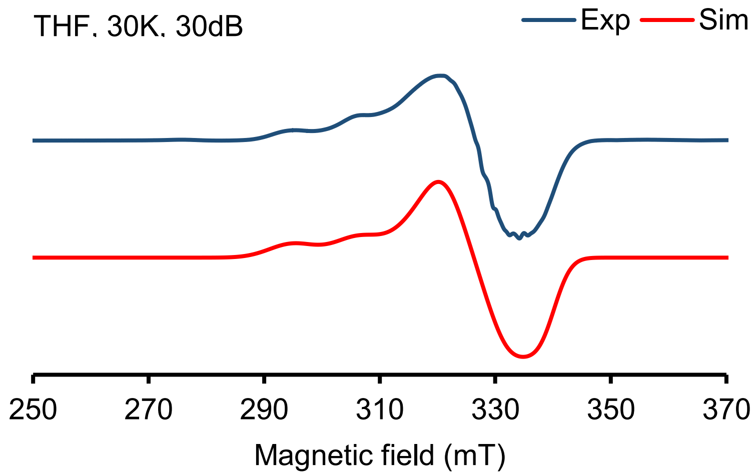

Figure S9. (A) X-band EPR spectrum [TBA]PDACu^{II}-Ph(CF$_3$)$_2$ (frozen THF, 1 mM), g_1 = 2.03 g_2 = 2.09, g_3 = 2.17, A_1= 85 MHz, A_2= 35 MHz, A_3= 340 MHz. The experimental spectrum is shown in blue, and the simulation is depicted in red.

6. Derivation of Rate Law

PT-ET (PT-limiting) pathway:

$$\mathbf{LH}Cu_3(II,II,II)(O)^{5+} \underset{k_{PT-1}}{\overset{k_{PT1}}{\rightleftharpoons}} \mathbf{L}Cu_3(II,II,II)(O\mathbf{H})^{5+} \quad \text{(slow)} \quad ....(1)$$

$$\mathbf{L}Cu_3(II,II,II)(O\mathbf{H})^{5+} + Cu^{II} \xrightarrow{k_{ET2}} \mathbf{L}Cu_3(II,II,I)(O\mathbf{H})^{4+} + Cu^{III} \quad \text{(fast)} \quad .....(2)$$

$$\mathbf{L}Cu_3(II,II,I)(O\mathbf{H})^{4+} + Cu^{II} \xrightarrow{k_{ET3}} \mathbf{L}Cu_3(II,I,I)(O\mathbf{H})^{3+} + Cu^{III} \quad \text{(fast)} \quad .....(3)$$

Steady state treatement on $\mathbf{L}Cu_3(II,II,II)(O\mathbf{H})^{5+}$

$$0 = k_{PT1}\,[\mathbf{LH}Cu_3(II,II,II)(O)]^{5+} - k_{PT-1}\,[\mathbf{L}Cu_3(II,II,II)(O\mathbf{H})]^{5+} - k_{ET1}\,[\mathbf{L}Cu_3(II,II,II)(O\mathbf{H})]^{5+}[Cu^{II}]_0$$

$$[LCu_3(II,II,I)(OH)]^{5+} = \frac{k_{PT1}\,[\mathbf{LH}Cu_3(II,II,II)(O)]^{5+}}{k_{PT-1} + k_{ET1}[Cu^{II}]_0}$$

Each PT event in step (2) rapidly drains to an additional Cu^{III} *in step* (3).

Hence, for two Cu^{III} *produced,*

$$\frac{d\,[Cu^{III}]}{dt} = \frac{2\,k_{PT1}k_{ET1}\,[Cu^{II}]_0}{k_{PT-1} + k_{ET1}[Cu^{II}]_0}\,[\mathbf{LH}Cu_3(II,II,II)(O)]^{5+}$$

Since the subsequent ET_1 *is much faster than* PT_{-1},

$$k_{ET1}[Cu^{II}]_0 >> k_{PT-1}$$

$$\frac{d\,[Cu^{III}]}{dt} = 2\,k_{PT1}\,[\mathbf{LH}Cu_3(II,II,II)(O)]^{5+}$$

For the ET-PT (ET-limiting) pathway,

$$\mathbf{LH}Cu_3(II,II,II)(O)^{5+} + Cu^{II} \underset{k_{ET-2}}{\overset{k_{ET2}}{\rightleftharpoons}} \mathbf{LH}Cu_3(II,II,I)(O)^{4+} + Cu^{III} \quad \text{(slow)} \quad ....(4)$$

$$\mathbf{LH}Cu_3(II,II,I)(O)^{4+} \xrightarrow{k_{PT2}} \mathbf{L}Cu_3(II,II,I)(O\mathbf{H})^{4+} \quad \text{(fast)} \quad .....(5)$$

$$\mathbf{L}Cu_3(II,II,I)(O\mathbf{H})^{4+} + Cu^{II} \xrightarrow{k_{ET3}} \mathbf{L}Cu_3(II,I,I)(O\mathbf{H})^{3+} + Cu^{III} \quad \text{(fast)} \quad .....(6)$$

Steady state treatement on $\mathbf{LH}Cu_3(II,II,I)(O)^{4+}$

$$0 = k_{ET2}\,[\mathbf{LH}Cu_3(II,II,II)(O)]^{5+}\,[Cu^{II}]_0 - (k_{ET\text{-}2}[Cu^{III}] + k_{PT2})\,[\mathbf{LH}Cu_3(II,II,I)(O)]^{4+}$$

$$[\mathbf{LH}Cu_3(II,II,I)(O)]^{4+} = \frac{k_{ET2}\,[\mathbf{LH}Cu_3(II,II,II)(O)]^{5+}\,[Cu^{II}]_0}{(k_{ET\text{-}2}[Cu^{III}] + k_{PT2})}$$

Each PT event in step (5) rapidly drains to an additional Cu^{III} *in step (6).*

Hence, for two Cu^{III} *produced,*

$$\frac{d\,[Cu^{III}]}{dt} = \frac{2\,k_{ET2}k_{PT2}\,[Cu^{II}]_0}{k_{ET\text{-}2}[Cu^{III}] + k_{PT2}}\,[\mathbf{LH}Cu_3(II,II,II)(O)]^{5+}$$

when, $k_{ET\text{-}2}[Cu^{III}] << k_{PT2}$

$$\frac{d\,[Cu^{III}]}{dt} = 2\,k_{ET2}\,[Cu^{II}]_0\,[\mathbf{LH}Cu_3(II,II,II)(O)]^{5+}$$

Finally, for the CPET pathway,

$$\mathbf{LH}Cu_3(II,II,II)(O)^{5+} + Cu^{II} \xrightarrow{k_{CPET}} \mathbf{L}Cu_3(II,II,I)(O\mathbf{H})^{4+} + Cu^{III} \quad \text{(slow)} \quad ....(7)$$

$$LCu_3(II,II,I)(O\mathbf{H})^{4+} + Cu^{II} \xrightarrow{k_{ET3}} LCu_3(II,I,I)(O\mathbf{H})^{3+} + Cu^{III} \quad \text{(fast)} \quad ......(8)$$

Hence, for two Cu^{III} produced,

$$\frac{d\,[Cu^{III}]}{dt} = 2\,k_{CPET}[Cu^{II}]_0\,[\mathbf{LH}Cu_3(II,II,II)(O)]^{5+}$$

Thus, the overall rate of expression for the formation of Cu^{III} *is*

$$\frac{d\,[Cu^{III}]}{dt} = 2\,(k_{ET2}[Cu^{II}]_0 + k_{CPET}[Cu^{II}]_0 + k_{PT1})\,[\mathbf{LH}Cu_3(II,II,II)(O)]^{5+}$$

7. UV-Vis spectroscopy and kinetic simulation details

Kinetic study of the reduction of [LHCu$_3$(II,II,II)(O)](PF$_6$)$_5$ with [TBA]PDACu^{II}-Ph at -70 ^{o}C

$$[\mathbf{LH}Cu_3(II,II,II)(O)]^{5+} + [PDACu^{II}\text{–}Ph]^{-} \xrightarrow[-70\ °C]{Acetone} [\mathbf{L}Cu_3(II,I,I)(O\mathbf{H})]^{3+} + [PDACu^{III}\text{–}Ph]$$

1 equiv.   10 equiv.

In a glovebox, an acetone solution (2.5 mL) of [**LH**Cu_3(II,II,II)(O)](PF_6)$_5$ (1.0 mg, 0.6 μmol) was transferred to a quartz cuvette equipped with a septum under N_2. The cuvette was sealed and transferred to the UV-Vis spectrometer, where it was cooled to −70 °C. Under nitrogen protection, an acetone solution (0.5 mL) of [**TBA**]PDACu^{II}-Ph (6.0 mg, 6 μmol, 10 equiv.) was injected into the cuvette using a 1-mL syringe with vigorous stirring. The reaction progress was monitored by UV–vis at −70 °C, with spectra recorded every 6 seconds (scan interval) over a total period of 15 minutes.

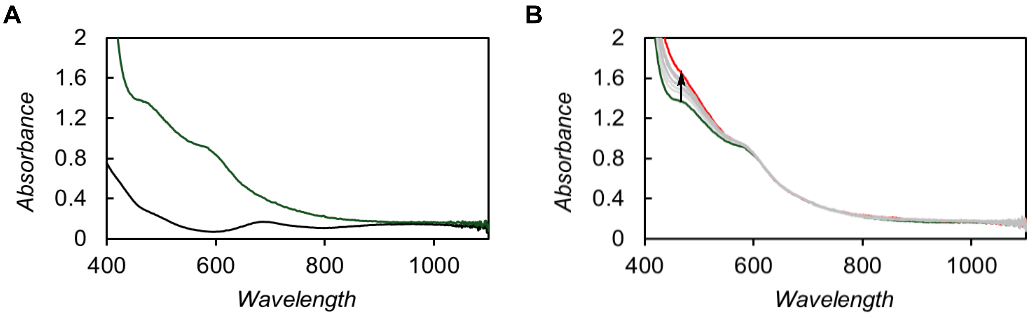

Figure S11. (A) In-situ UV-vis spectra of the reaction of **LH**Cu_3(II,II,II)(O)(PF_6)$_5$ with 10 equiv. of [TBA]PDACu^{II}-Ph at −70 °C. (Step I). (B) In-situ UV-vis spectra of the formation of PDACu^{III}-Ph over the course of reaction.

Kinetic study of the reduction of [LHCu$_3$(II,II,II)(O)](PF$_6$)$_5$ with [TBA]PDACu^{II}-PhCF$_3$ at -80 ^{o}C to -50 ^{o}C

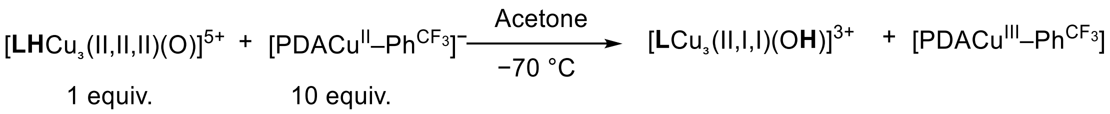

In a glovebox, an acetone solution (2.5 mL) of [**LH**Cu_3(II,II,II)(O)](PF_6)$_5$ (1.0 mg, 0.6 μmol) was transferred to a quartz cuvette equipped with a septum under N_2. The cuvette was sealed and transferred to the UV-Vis spectrometer. Under nitrogen protection, an acetone solution (0.5 mL) of [**TBA**]PDACu^{II}-PhCF$_3$ (6.4 mg, 6 μmol, 10 equiv.) was injected into the cuvette using a 1-mL syringe with vigorous stirring. The progress of the reaction was monitored by UV–Vis spectroscopy at −50 °C, −60 °C, −70 °C, and −80 °C, with spectra recorded at 30 s intervals at each temperature.

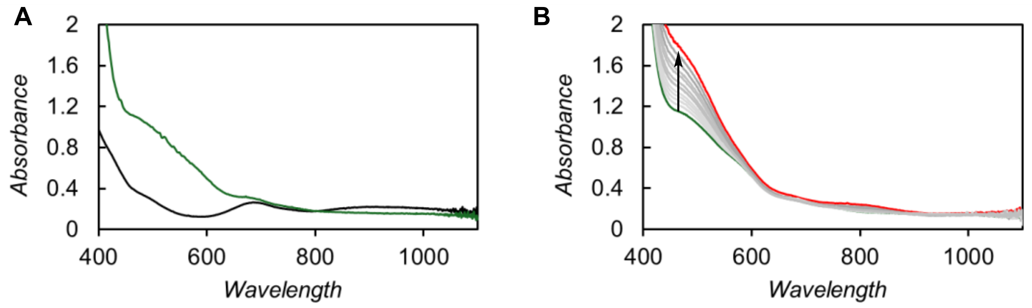

Figure S12. In-situ UV-vis spectra of the reaction of **LH**Cu_3(II,II,II)(O)$(PF_6)_5$ with 10 equiv. of [TBA]PDACu^{II}-$PhCF_3$ at −80 °C. (Step I). (B) In-situ UV-vis spectra of the formation of PDACu^{III}-$PhCF_3$ over the course of reaction.

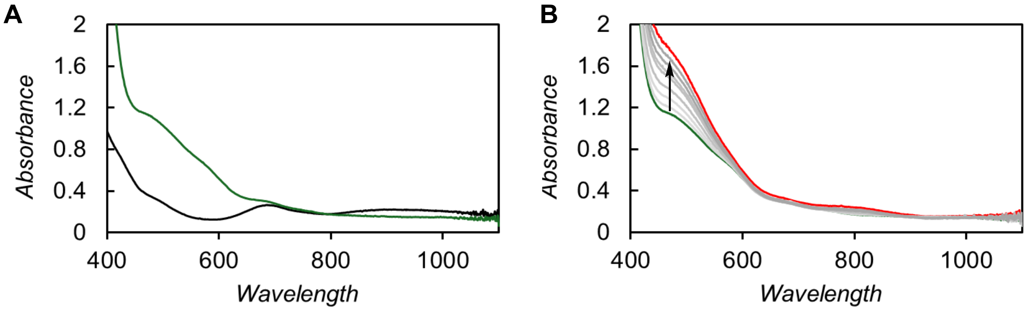

Figure S13. In-situ UV-vis spectra of the reaction of **LH**Cu_3(II,II,II)(O)$(PF_6)_5$ with 10 equiv. of [TBA]PDACu^{II}-$PhCF_3$ at −70 °C. (Step I). (B) In-situ UV-vis spectra of the formation of PDACu^{III}-$PhCF_3$ over the course of reaction.

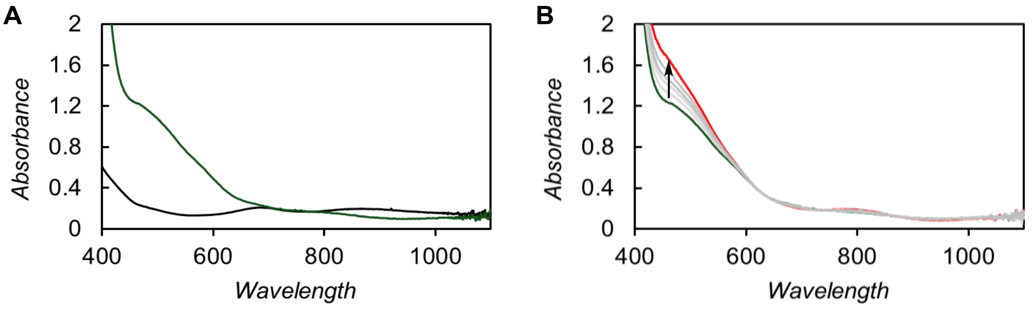

Figure S14. In-situ UV-vis spectra of the reaction of **LH**Cu_3(II,II,II)(O)$(PF_6)_5$ with 10 equiv. of [TBA]PDACu^{II}-$PhCF_3$ at −60 °C. (Step I). (B) In-situ UV-vis spectra of the formation of PDACu^{III}-$PhCF_3$ over the course of reaction.

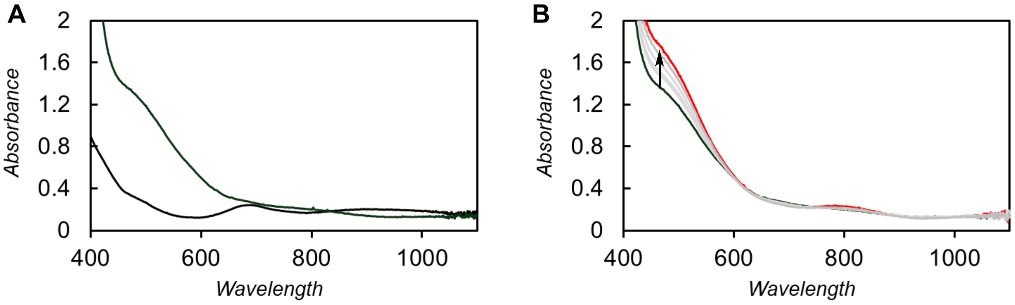

Figure S15. In-situ UV-vis spectra of the reaction of **LH**Cu_3(II,II,II)(O)(PF_6)$_5$ with 10 equiv. of [TBA]PDACu^{II}-$PhCF_3$ at −50 °C. (Step I). (B) In-situ UV-vis spectra of the formation of PDACu^{III}-$PhCF_3$ over the course of reaction.

Kinetic study of the reduction of [LHCu$_3$(II,II,II)(O)](PF$_6$)$_5$ with [TBA]PDACu^{II}-Ph(CF$_3$)$_2$ at -70 °C

$$[\mathbf{LH}Cu_3(II,II,II)(O)]^{5+} + [PDACu^{II}\text{–}Ph^{(CF_3)_2}]^- \xrightarrow[-70\ °C]{Acetone} [\mathbf{L}Cu_3(II,II,I)(O\mathbf{H})]^{4+} + [PDACu^{III}\text{–}Ph^{(CF_3)_2}]$$

1 equiv. 10 equiv.

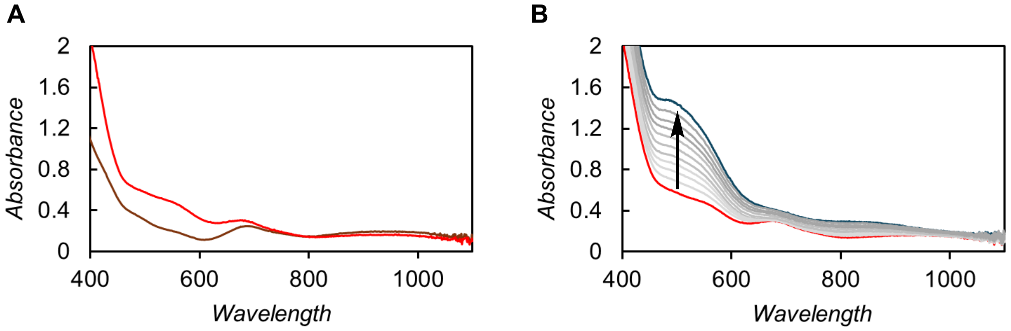

Figure S16. (A) In-situ UV-vis spectra of the reaction of **LH**Cu_3(II,II,II)(O)(PF_6)$_5$ with 10 equiv. of [TBA]PDACu^{II}-Ph(CF_3)$_2$ at −70 °C. (Step I). (B) In-situ UV-vis spectra of the formation of PDACu^{II}-Ph(CF_3)$_2$ with over the course of reaction.

In glovebox, an acetone solution (2.5 mL) of [**LH**Cu_3(II,II,II)(O)](PF_6)$_5$ (1 mg, 0.6 µmol) was transferred to a quartz cuvette equipped with a septum under N_2. The cuvette was sealed and transferred to the UV-Vis spectrometer, where it was cooled to −70 °C Under nitrogen protection, an acetone solution (0.5 mL) of [**TBA**]PDACu^{II}-Ph(CF_3)$_2$ (6.8 mg, 6 µmol, 10 equiv.) was injected into the cuvette using a 1-mL syringe with vigorous stirring. The progress of the reaction was monitored with UV-vis at −70 °C at an interval of 1 minute.

Kinetic study of the reduction of [LHCu$_3$(II,II,II)(O)](PF$_6$)$_5$ with bis(benzene)chromium(0) at -70 °C

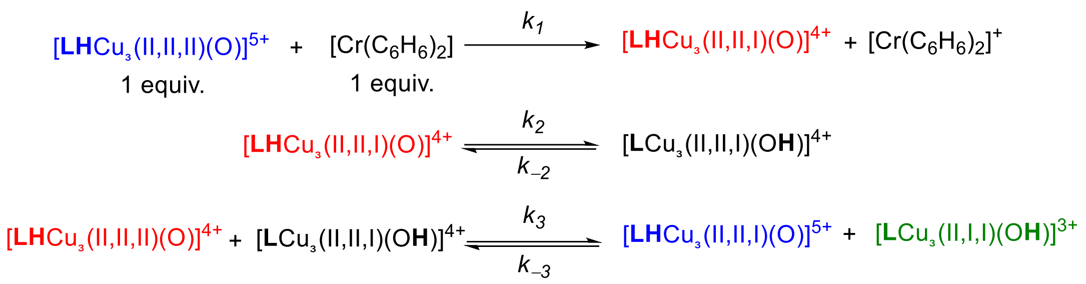

To investigate the PCET mechanism using a reductant slightly weaker than cobaltocene (E° = −1.33 V vs Fc/Fc$^+$),[3] the reduction of [**LH**Cu$_3$(II,II,II)(O)]$^{5+}$ was examined using bis(benzene)chromium(0) (E° = −1.15 V vs Fc/Fc$^+$) as the reductant. In a glovebox, an acetone solution (2.9 mL) of [**LH**Cu$_3$(II,II,II)(O)](PF$_6$)$_5$ (2 mg, 1.2 μmol) was transferred to a quartz cuvette equipped with a septum under N$_2$. The cuvette was sealed and transferred to the UV-Vis spectrometer. Bis(benzene)chromium(0) (2.5 mg, 12 μmol, 10 eq.) was dissolved in acetone (1 mL), and 0.1 mL of the solution (1.2 μmol, 1 eq.) was injected to the UV-vis cuvette. The progress of the reaction was monitored with UV-vis at −70 °C (15 s interval, Figure S17). Kinetic simulations and numerical fitting of the time-resolved UV–Vis data were performed using KinTek Explorer. The experimental traces were globally fitted using a mechanistically constrained kinetic model that incorporates sequential electron- and proton-transfer steps. Rate constants for individual elementary steps were optimized to reproduce the full temporal and spectral evolution of the system, while enforcing thermodynamic self-consistency and microscopic reversibility (Table S9). The resulting simulations accurately capture the observed kinetics and support the proposed reaction mechanism.

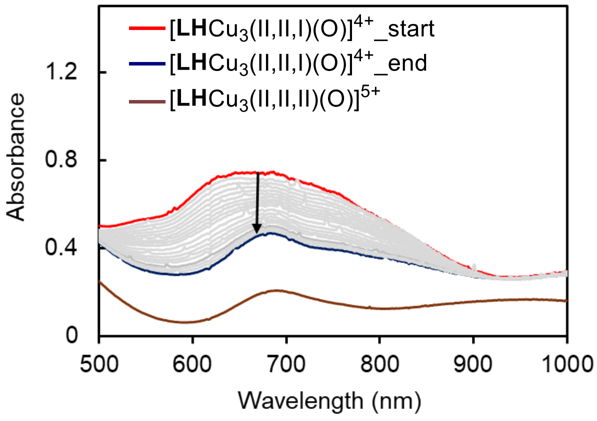

Figure S17. UV-vis spectrum of the reaction between [**LH**Cu$_3$(II,II,II)(O)](PF$_6$)$_5$ and bis(benzene)chromium(0) in acetone at −70 °C.

Kinetic study of the reduction of [LHCu$_3$(II,II,II)(O)](PF$_6$)$_5$ with decamethylferrocene at -70 ^{o}C

$$[\mathbf{LH}Cu_3(II,II,II)(O)]^{5+} + [Fe(C_5(CH_3)_5)_2] \xrightarrow[-70\ °C]{\text{Acetone}} [\mathbf{L}Cu_3(II,II,I)(O\mathbf{H})]^{4+} + [Fe(C_5(CH_3)_5)_2]^{+}$$

1 equiv. 10 equiv.

To investigate the PCET mechanism using a reductant similar to [**TBA**]PDACu^{II}-Ph(CF$_3$)$_2$ (E° = −0.46 V vs Fc/Fc$^+$), the reduction of [**LH**Cu$_3$(II,II,II)(O)]$^{5+}$ was examined using decamethylferrocene (Me$_{10}$Fc) (E° = −0.48 V vs Fc/Fc$^+$). However, experiments with Me$_{10}$Fc were conducted at one-fourth the concentration used for [**TBA**]PDACu^{II}–Ph(CF$_3$)$_2$ due to the limited solubility of Me$_{10}$Fc. In a glovebox, an acetone solution (2.5 mL) of [**LH**Cu$_3$(II,II,II)(O)](PF$_6$)$_5$ (0.25 mg, 0.15 μmol) was transferred to a quartz cuvette equipped with a septum under N$_2$. The cuvette was sealed and transferred to the UV-Vis spectrometer, where it was cooled to −70 °C Under nitrogen protection, an acetone solution (0.5 mL) of Me$_{10}$Fc (0.5 mg, 1.5 μmol, 10 equiv.) was injected into the cuvette using a 1-mL syringe with vigorous stirring. The progress of the reaction was monitored with UV-vis at −70 °C at an interval of 30 seconds.

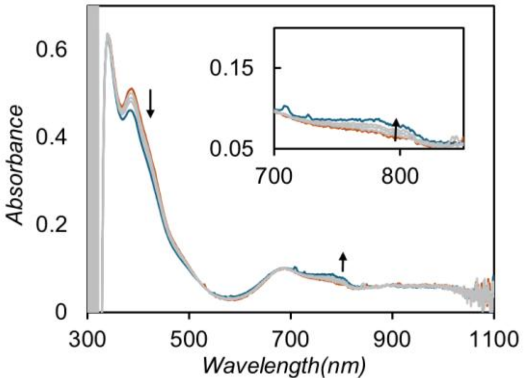

Figure S18. UV-vis spectrum of the reaction between [**LH**Cu$_3$(II,II,II)(O)](PF$_6$)$_5$ and [Fe(C$_5$(CH$_3$)$_5$)$_2$] in acetone at −70 °C. Inset: Formation of ferrocenium hexafluorophosphate at 785 and 805 nm over the course of the reaction.

Kinetic study of the oxidation of [LCu$_3$(II,II,I)(OH)](PF$_6$)$_5$ with tris(4-bromophenyl)ammoniumyl hexafluorophosphate (magic blue) from -75 ^{o}C to -60 ^{o}C

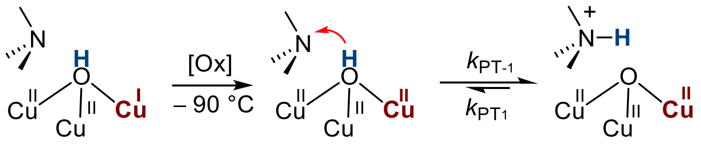

In a glovebox, an acetone solution (2.8 mL) of [**L**Cu_3(II,II,I)(O**H**)](PF_6)$_4$ (2.5 mg, 1.64 μmol) was transferred to a quartz cuvette equipped with a septum under N_2. The cuvette was sealed and transferred to the UV-Vis spectrometer. Under nitrogen protection, an acetone solution (1 mL) of magic blue (10.3 mg, 16.4 μmol, 10 equiv.) was prepared, and 0.1 ml (1equiv.) was injected into the cuvette using a 1-mL syringe with vigorous stirring. The progress of the reaction was monitored with UV-vis at −60 °C, −65 °C, −70 °C, and −75 °C, respectively, with spectra recorded at 6-second intervals at each temperature.

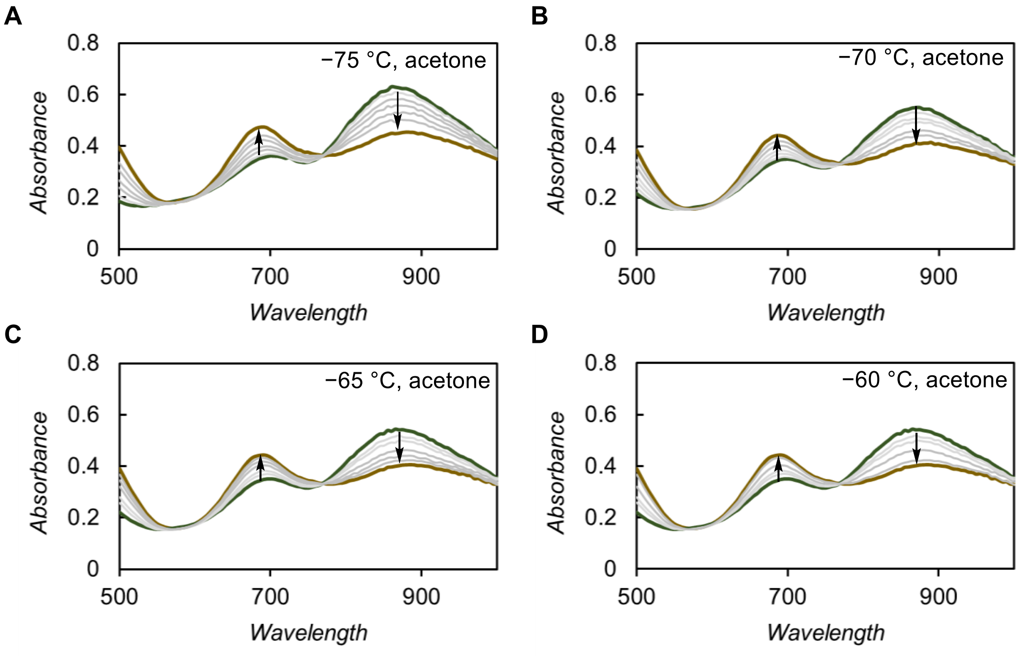

Figure S19. UV-vis spectrum of the reaction between [**L**Cu_3(II,II,I)(O**H**)](PF_6)$_4$ and Magic Blue in acetone at −75 °C (A), −70 °C (B), −65 °C (C), and −60 °C (D).

Temperature	Trial	k_{PT-1}	k_{PT-1}_avg	k_{PT-1}_std. dev.	k_{PT1}	k_{PT1}_avg	k_{PT1}_std. dev.
	Set 1	1.10E-05			7.86E-04		
-75	Set 2	2.12E-05	1.37E-05	6.56E-06	1.51E-03	9.80E-04	4.66E-04
	Set 3	8.96E-06			6.40E-04		
	Set 1	2.60E-05			1.86E-03		
-70	Set 2	2.90E-05	2.83E-05	8.96E-07	2.07E-03	2.02E-03	6.08E-05
	Set 3	2.99E-05			2.14E-03		
	Set 1	7.65E-05			5.46E-03		
-65	Set 2	6.62E-05	6.97E-05	5.86E-06	4.73E-03	4.11E-03	4.16E-04
	Set 3	6.65E-05			4.75E-03		
	Set 1	1.05E-04			7.50E-03		
-60	Set 2	2.14E-04	1.39E-04	6.47E-05	1.53E-02	9.96E-03	4.63E-03
	Set 3	9.92E-05			7.09E-03		

Table S1. Summary of rate constants from kinetic simulation for the intramolecular proton transfer between [**L**Cu_3(II,II,II)(O**H**)]$^{5+}$ and [**LH**Cu_3(II,II,II)(O)]$^{5+}$ at different temperatures. The upper and lower limits for rate constant were derived from the threshold of Chi^2_{min}/ Chi^2 = 0.9.. The values of k_{PT-1} and k_{PT1} are dependent on the estimated K_{eq}.[7]

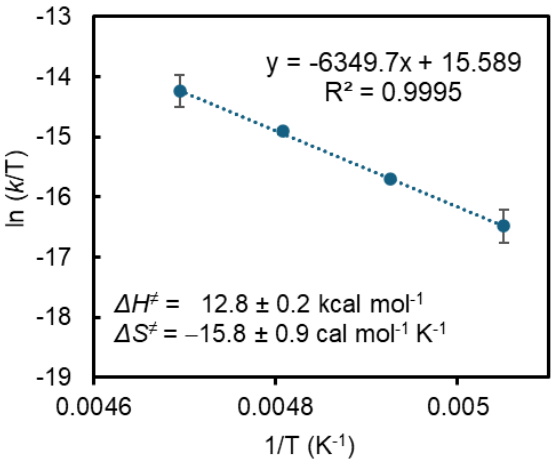

Figure S20. Eyring plot of the Intramolecular proton transfer from [**LH**Cu_3(II,II,II)(O)]$^{5+}$ to [**L**Cu_3(II,II,II)(O**H**)]$^{5+}$.

Kinetic modelling and simulation parameters

Kinetic modeling and numerical data fitting were performed using *KinTek* Explorer (version 11.0.1).[8,9] The model employed in this work is a modified version of the previously established *KinTek* model for the reduction of [**LH**Cu_3(II,II,II)(O)]$^{5+}$ by cobaltocene.[3] The modifications account for the competitive reduction of [**LH**Cu_3(II,II,II)(O)]$^{5+}$ by excess reductant (10 equiv.), including [TBA]PDACu^{II}–R and decamethylferrocene ($Me_{10}Fc$), through three distinct proton-coupled electron transfer (PCET) pathways.

The analysis focused on wavelength regions of the time-resolved UV–Vis spectra where the absorbance changes arise predominantly from species involved in the PCET process and contributions from other components are minimal. For reactions with [TBA]PDACu^{II}–R, the 400–600 nm region was used to monitor formation of oxidized PDACu^{III}–R species. For reactions with $Me_{10}Fc$, the 340–500 nm region was analyzed to track the conversion of [**LH**Cu_3(II,II,II)(O)]$^{5+}$ to [**L**Cu_3(II,II,I)(O**H**)]$^{4+}$. The workflow comprised three stages:

(1) Spectral rank analysis by singular value decomposition (SVD) to determine the number of kinetically distinct intermediates,
(2) Construction of a mechanistically consistent PCET model, and
(3) Numerical fitting of the kinetic traces under thermodynamic and experimental constraints.

Spectra preprocessing and SVD analysis

UV–Vis datasets were first baseline-corrected prior to kinetic analysis. For experiments involving $Me_{10}Fc$, the background absorbance from excess reductant under pseudo-first-order conditions was removed by subtracting a time-zero reference spectrum recorded at an identical $Me_{10}Fc$ concentration, yielding $\Delta A(\lambda,t)$. After baseline correction, the relevant wavelength window was retained for analysis.

The processed absorbance–time matrices (λ vs t) were decomposed via SVD according to

$$A(\lambda,t) = U\,S\,V^T$$

where U represents the basis spectra, V the corresponding time-dependent amplitude vectors, and S the singular values. The logarithmic plot of singular values revealed two dominant components above the noise floor, consistent with the presence of two kinetically significant intermediates. Reconstruction of the dataset using these two components reproduced the full spectral evolution, validating the use of a two-state kinetic model for subsequent fitting.

Kinetic model description

The first pathway is a stepwise proton-first mechanism (PT–ET), in which intramolecular proton transfer (PT_1) from [**LH**Cu_3(II,II,II)(O)]$^{5+}$ generates the hydroxo intermediate [**L**Cu_3(II,II,II)(OH)]$^{5+}$ followed by outer-sphere electron transfer (ET_1) to afford [**L**Cu_3(II,II,I)(OH)]$^{4+}$.

The second pathway is a stepwise electron-first mechanism (ET–PT), involving initial electron transfer (ET_2) from [**LH**Cu_3(II,II,II)(O)]$^{5+}$ to yield [**LH**Cu_3(II,II,I)(O)]$^{4+}$, followed by intramolecular proton transfer from the **LH** scaffold (PT_2) to furnish [**L**Cu_3(II,II,I)(O**H**)]$^{4+}$. The third pathway involves a concerted electron–proton transfer (CEPT), wherein both the electron and proton tunnel through the same transition state in a single kinetic step, directly converting [**LH**Cu_3(II,II,II)(O)]$^{5+}$ to [**L**Cu_3(II,II,I)(O**H**)]$^{4+}$.

In the case of [TBA]PDACu^{II}–Ph and [TBA]PDACu^{II}–PhCF_3, the resulting species [**L**Cu_3(II,II,I)(O**H**)]$^{4+}$ undergoes a subsequent one-electron reduction to form [**L**Cu_3(II,I,I)(O**H**)]$^{3+}$, completing the overall reductive sequence.

$$[\mathbf{LH}Cu_3(II,II,II)(O)]^{5+} \underset{k_{-PT1}}{\overset{k_{PT1}}{\rightleftharpoons}} [\mathbf{L}Cu_3(II,II,II)(OH)]^{5+} \quad (1)$$

$$[\mathbf{L}Cu_3(II,II,II)(OH)]^{5+} + Cu^{II} \text{ or } Fe^{II} \underset{k_{-ET1}}{\overset{k_{ET1}}{\rightleftharpoons}} [\mathbf{L}Cu_3(II,II,I)(OH)]^{4+} + Cu^{III} \text{ or } Fe^{III} \quad (2)$$

$$[\mathbf{LH}Cu_3(II,II,II)(O)]^{5+} + Cu^{II} \text{ or } Fe^{II} \underset{k_{-ET2}}{\overset{k_{ET2}}{\rightleftharpoons}} [\mathbf{LH}Cu_3(II,II,I)(O)]^{4+} + Cu^{III} \text{ or } Fe^{III} \quad (3)$$

$$[\mathbf{LH}Cu_3(II,II,I)(O)]^{4+} \underset{k_{-PT2}}{\overset{k_{PT2}}{\rightleftharpoons}} [\mathbf{L}Cu_3(II,II,I)(OH)]^{4+} \quad (4)$$

$$[\mathbf{LH}Cu_3(II,II,II)(O)]^{5+} + Cu^{II} \text{ or } Fe^{II} \overset{k_{PCET}}{\rightleftharpoons} [\mathbf{L}Cu_3(II,II,I)(OH)]^{4+} + Cu^{III} \text{ or } Fe^{III} \quad (5)$$

$$[\mathbf{L}Cu_3(II,II,I)(OH)]^{4+} + Cu^{II} \xrightarrow{k_{ET3}} [\mathbf{L}Cu_3(II,I,I)(OH)]^{3+} + Cu^{III} \quad (6)$$

Scheme S1. Summary of different PCET pathways for the reduction of [**LH**Cu_3(II,II,II)(O)]$^{5+}$ with [TBA]PDACu^{II}–R as reductants.

Approximate rate constants were obtained from single-exponential fits of the dominant kinetic phases, which served as starting estimates for global fitting. All datasets were then

simultaneously optimized across temperatures by minimizing the global χ^2.

Parameter constraints

To ensure physical realism and thermodynamic self-consistency, key kinetic parameters were fixed or linked according to independently determined experimental quantities:

- Intramolecular proton-transfer steps (PT_1 and PT_2) were treated as fast, reversible equilibria. Their forward and reverse rate constants were locked based on experimentally measured proton-transfer rates.[3] The ratios of these rate constants were constrained to reproduce the experimentally established equilibrium constants ($K_{eq,\,PT1}$ and $K_{eq,\,PT2}$) that describe proton movement within the [**LH**Cu_3(II,II,II)(O)]$^{5+}$ core and the pendant TREN amine site, respectively.[7]
- Electron-transfer steps (ET_1 and ET_2). The electron-transfer reactions were constrained by equilibrium constants derived from the difference in redox potentials ($\Delta E°$) between the tricopper complex and the corresponding reductant ([TBA]PDACu^{II}–R or Me_{10}Fc).
- During fitting, the forward ET rate constants were optimized, while the reverse ET rate constants were automatically computed from the corresponding $K_{eq,\,ET}$ values, preserving full thermodynamic closure among the redox steps.
- The CEPT process was modeled as a single irreversible kinetic event, representing simultaneous electron and proton transfer through a common transition state. Its microscopic reversibility was enforced by linking the reverse rate constant to the overall free-energy change ($\Delta G°_{CPET}$) calculated from the redox potentials and the proton-transfer equilibrium constant.
- For [TBA]PDACu^{II}–Ph and [TBA]PDACu^{II}–PhCF_3, the downstream ET_3 step was included for thermodynamic completeness. The electron-transfer rate was found to be non-identifiable, as its precursor species are not significantly populated within the experimental time window. ET_3 was therefore fixed to an effectively instantaneous rate ($k_{ET3} = 1 \times 10^6$ $M^{-1}s^{-1}$). Varying this value over several orders of magnitude did not affect the simulated results.
- To ensure microscopic reversibility, $\Delta G°$ of the three PCET pathways were enforced to the same, ensuring internal consistency among forward and reverse rate constants across all pathways.

Model validation

The quality of the kinetic fits was evaluated by analysis of residual distributions, χ^2 statistics, and goodness-of-fit parameters. FitSpace Explorer tools within *KinTek* Explorer were used to determine confidence intervals and assess parameter identifiability. Comparative fitting using reduced models—such as PT–ET-only, ET–PT-only, or CEPT-only—produced significantly higher χ^2 values and failed to reproduce the full spectral and temporal evolution observed experimentally. In contrast, the complete three-pathway model provided a statistically superior fit and yielded parameter values consistent with independent electrochemical and thermochemical measurements. Together, these results confirm that the chosen kinetic model accurately captures the mechanistic complexity of the tricopper-mediated PCET reduction.

	Rates	$k_{PT1}{}^{a}(s^{-1})$	$k_{PT-1}{}^{a}(s^{-1})$	$k_{ET1}{}^{c}(M^{-1}s^{-1})$	$k_{ET-1}{}^{c}(M^{-1}s^{-1})$	$k_{ET2}{}^{d}(M^{-1}s^{-1})$	$k_{ET-2}{}^{d}(M^{-1}s^{-1})$	$k_{PT2}{}^{b}(s^{-1})$	$k_{PT-2}{}^{b}(s^{-1})$	$k_{CPET}(M^{-1}s^{-1})$	$k_{CPET-1}(M^{-1}s^{-1})$	$k_{ET3}(M^{-1}s^{-1})$
	Upper	-	-	3.65E+05	-	8.73E-03	-	-	-	1.56E+00	-	-
Trial 1	best fit	2.83E-05	1.98E-03	2.92E+05	2.03E-07	7.00E-03	4.00E-04	3.30E-03	2.80E-11	1.25E+00	6.06E-10	1.00E+06
	Lower	-	-	2.34E+05	-	5.59E-03	-	-	-	9.98E-01	-	-
	Upper	-	-	3.41E+05	-	7.32E-02	-	-	-	1.27E+00	-	-
Trial 2	best fit	2.83E-05	1.98E-03	2.73E+05	2.77E-07	5.85E-02	4.90E-04	3.30E-03	2.80E-11	1.01E+00	7.20E-11	1.00E+06
	Lower	-	-	2.18E+05	-	4.68E-02	-	-	-	8.11E-01	-	-
	Upper	-	-	2.97E+05	-	1.19E-02	-	-	-	1.24E+00	-	-
Trial 3	best fit	2.83E-05	1.98E-03	2.72E+05	2.07E-07	1.09E-02	6.84E-04	3.30E-03	2.80E-11	1.14E+00	6.06E-10	1.00E+06
	Lower	-	-	2.54E+05	-	1.02E-02	-	-	-	1.06E+00	-	-
Avergae		2.83E-05	1.98E-03	2.79E+05	2.29E-07	2.55E-02	5.25E-04	3.30E-03	2.80E-11	1.13E+00	4.28E-10	-
Std. deviation		-	-	9.20E+03	3.40E-08	2.34E-02	1.19E-04	-	-	9.81E-02	2.52E-10	-

Table S2. Summary of rate constants from kinetic simulation for the reaction between [**LH**Cu_3(II,II,II)(O)]$^{5+}$ and [TBA]PDACu^{II}–Ph at -70 °C. The upper and lower limits for the rate constant were derived from the threshold of Chi^2_{min}/ Chi^2 = 0.9. [a] values that are dependent on k_{PT1}. [b] values that are dependent on $k_{PT2.}$ The values of k_{PT-1} and k_{PT-2} are dependent on k_{PT1} and $k_{PT2,}$ respectively, from the estimated $K_{eq}= k_{PT1}/ k_{PT-1}$ and $K_{eq}= k_{PT2}/ k_{PT-2}$ previously reported.[3,7] [c] values that are dependent on k_{ET1}. [d] values that are dependent on $k_{ET2.}$ The values of k_{ET-1} and k_{ET-2} are dependent on k_{ET1} and k_{ET2} respectively, from the estimated K_{eq}, where RT lnK_{eq} = –nFΔE°, where, $\Delta E^{o} = E^{o}_{[\mathbf{LH}Cu3(II,II,II)(O)]5+} - E^{o}_{PDACuII-Ph}$. k_{ET3} was fixed to an effectively instantaneous rate (k_{ET3} = 1 × 10^{6} $M^{-1}s^{-1}$). Varying this value over several orders of magnitude did not affect the simulated results.

	Rates	$k_{PT1}{}^{a}(s^{-1})$	$k_{PT-1}{}^{a}(s^{-1})$	$k_{ET1}{}^{c}(M^{-1}s^{-1})$	$k_{ET-1}{}^{c}(M^{-1}s^{-1})$	$k_{ET2}{}^{d}(M^{-1}s^{-1})$	$k_{ET-2}{}^{d}(M^{-1}s^{-1})$	$k_{PT2}{}^{b}(s^{-1})$	$k_{PT-2}{}^{b}(s^{-1})$	$k_{CPET}(M^{-1}s^{-1})$	$k_{CPET-1}(M^{-1}s^{-1})$	$k_{ET3}(M^{-1}s^{-1})$
	Upper	-	-	1.80E+04	-	2.77E-03	-	-	-	8.60E-02	-	-
Trial 1	best fit	5.42E-06	3.79E-04	1.65E+04	1.63E-04	2.54E-03	2.07E-01	1.00E-03	8.47E-12	7.89E-02	5.45E-08	1.00E+06
	Lower	-	-	1.54E+04	-	2.38E-03	-	-	-	7.38E-02	-	-
	Upper	-	-	1.78E+04	-	8.96E-04	-	-	-	8.22E-02	-	-
Trial 2	best fit	5.42E-06	3.79E-04	1.58E+04	1.69E-04	8.21E-04	7.01E-02	1.00E-03	8.47E-12	7.54E-02	5.47E-08	1.00E+06
	Lower	-	-	1.53E+04	-	7.69E-04	-	-	-	7.05E-02	-	-
	Upper	-	-	1.10E+04	-	1.04E-03	-	-	-	7.20E-02	-	-
Trial 3	best fit	5.42E-06	3.79E-04	1.00E+04	1.63E-04	9.51E-04	9.48E-02	1.00E-03	8.47E-12	6.59E-02	5.56E-08	1.00E+06
	Lower	-	-	9.15E+03	-	8.69E-04	-	-	-	6.01E-02	-	-
Avergae		5.42E-06	3.79E-04	1.41E+04	1.65E-04	1.44E-03	1.24E-01	1.00E-03	8.47E-12	7.34E-02	5.49E-08	-
Std. deviation		-	-	3.57E+03	3.46E-06	9.57E-04	7.30E-02	-	-	6.73E-03	5.86E-10	-

Table S3. Summary of rate constants from kinetic simulation for the reaction between [**LH**Cu_3(II,II,II)(O)]$^{5+}$ and [TBA]PDACu^{II}–PhCF_3 at -80 ºC. The upper and lower limits for the rate constant were derived from the threshold of $Chi^2_{min}/ Chi^2 = 0.9$. [a] values that are dependent on k_{PT1}. [b] values that are dependent on $k_{PT2.}$ The values of k_{PT-1} and k_{PT-2} are dependent on k_{PT1} and $k_{PT2,}$ respectively, from the estimated $K_{eq}= k_{PT1}/ k_{PT-1}$ and $K_{eq}= k_{PT2}/ k_{PT-2}$ previously reported.[3,7] [c] values that are dependent on $k_{ET1.}$ [d] values that are dependent on $k_{ET2.}$ The values of k_{ET-1} and k_{ET-2} are dependent with k_{ET1} and $k_{ET2,}$ respectively, from the estimated K_{eq}, where $RT \ln K_{eq} = -nF\Delta E^o$, where, $\Delta E^o = E^o_{[\mathbf{LH}Cu3(II,II,II)(O)]5+} - E^o_{PDACuII–PhCF3}$. k_{ET3} was fixed to an effectively instantaneous rate ($k_{ET3} = 1 \times 10^6$ $M^{-1}s^{-1}$). Varying this value over several orders of magnitude did not affect the simulated results.

	Rates	$k_{PT1}{}^{a}(s^{-1})$	$k_{PT\text{-}1}{}^{a}(s^{-1})$	$k_{ET1}{}^{c}(M^{-1}s^{-1})$	$k_{ET\text{-}1}{}^{c}(M^{-1}s^{-1})$	$k_{ET2}{}^{d}(M^{-1}s^{-1})$	$k_{ET\text{-}2}{}^{d}(M^{-1}s^{-1})$	$k_{PT2}{}^{b}(s^{-1})$	$k_{PT\text{-}2}{}^{b}(s^{-1})$	$k_{CPET}(M^{-1}s^{-1})$	$k_{CPET\text{-}1}(M^{-1}s^{-1})$	$k_{ET3}(M^{-1}s^{-1})$
	Upper	-	-	2.34E+04	-	3.33E-03	-	-	-	9.61E-02	-	-
Trial 1	best fit	2.83E-05	1.98E-03	1.87E+04	1.54E-04	2.67E-03	1.81E-01	3.30E-03	2.80E-11	7.70E-02	4.43E-08	1.00E+06
	Lower	-	-	1.85E+04	-	2.63E-03	-	-	-	7.60E-02	-	-
	Upper	-	-	2.23E+04	-	2.29E-03	-	-	-	1.11E-01	-	-
Trial 2	best fit	2.83E-05	1.98E-03	1.71E+04	1.15E-04	1.91E-03	9.75E-02	3.30E-03	2.80E-11	1.02E-01	4.02E-08	1.00E+06
	Lower	-	-	1.83E+04	-	1.87E-03	-	-	-	9.08E-02	-	-
	Upper	-	-	1.75E+04	-	6.06E-03	-	-	-	7.99E-02	-	-
Trial 3	best fit	2.83E-05	1.98E-03	1.60E+04	1.39E-04	5.54E-03	3.96E-01	3.30E-03	2.80E-11	7.31E-02	4.43E-08	1.00E+06
	Lower	-	-	1.46E+04	-	5.06E-03	-	-	-	6.67E-02	-	-
Avergae		2.83E-05	1.98E-03	1.73E+04	1.36E-04	3.37E-03	2.25E-01	3.30E-03	2.80E-11	8.40E-02	4.29E-08	-
Std. deviation		-	-	1.36E+03	1.97E-05	1.91E-03	1.54E-01	-	-	1.57E-02	2.37E-09	-

Table S4. Summary of rate constants from kinetic simulation for the reaction between [**LH**Cu_3(II,II,II)(O)]$^{5+}$ and [TBA]PDACu^{II}–PhCF_3 at -70 ºC. The upper and lower limits for the rate constant were derived from the threshold of Chi^2_{min}/ Chi^2 = 0.9. [a] values that are dependent on k_{PT1}. [b] values that are dependent on $k_{PT2.}$ The values of $k_{PT\text{-}1}$ and $k_{PT\text{-}2}$ are dependent on k_{PT1} and $k_{PT2,}$ respectively, from the estimated $K_{eq}= k_{PT1}/ k_{PT\text{-}1}$ and $K_{eq}= k_{PT2}/ k_{PT\text{-}2}$ previously reported.[3,7] [c] values that are dependent on $k_{ET1.}$ [d] values that are dependent on $k_{ET2.}$ The values of $k_{ET\text{-}1}$ and $k_{ET\text{-}2}$ are dependent with k_{ET1} and $k_{ET2,}$ respectively, from the estimated K_{eq}, where RT lnK_{eq} = –nFΔE^o, where, $\Delta E^o = E^o_{[\mathbf{LH}Cu3(II,II,II)(O)]5+} - E^o_{PDACuII\text{–}PhCF3}$. k_{ET3} was fixed to an effectively instantaneous rate (k_{ET3} = 1×10^6 $M^{-1}s^{-1}$). Varying this value over several orders of magnitude did not affect the simulated results.

	Rates	$k_{PT1}{}^{a}(s^{-1})$	$k_{PT-1}{}^{a}(s^{-1})$	$k_{ET1}{}^{c}(M^{-1}s^{-1})$	$k_{ET-1}{}^{c}(M^{-1}s^{-1})$	$k_{ET2}{}^{d}(M^{-1}s^{-1})$	$k_{ET-2}{}^{d}(M^{-1}s^{-1})$	$k_{PT2}{}^{b}(s^{-1})$	$k_{PT-2}{}^{b}(s^{-1})$	$k_{CPET}(M^{-1}s^{-1})$	$k_{CPET-1}(M^{-1}s^{-1})$	$k_{ET3}(M^{-1}s^{-1})$
	Upper	-	-	2.07E+04	-	6.29E-03	-	-	-	2.13E-01	-	-
Trial 1	best fit	1.39E-04	9.73E-03	1.66E+04	6.93E-05	5.03E-03	1.73E-01	1.60E-02	1.36E-10	1.70E-01	4.96E-08	1.00E+06
	Lower	-	-	1.33E+04	-	4.03E-03	-	-	-	1.36E-01	-	-
	Upper	-	-	2.47E+04	-	7.03E-03	-	-	-	2.53E-01	-	-
Trial 2	best fit	1.39E-04	9.73E-03	2.26E+04	6.93E-05	6.43E-03	1.62E-01	1.60E-02	1.36E-10	2.32E-01	4.96E-08	1.00E+06
	Lower	-	-	2.06E+04	-	5.87E-03	-	-	-	2.11E-01	-	-
	Upper	-	-	1.26E+04	-	6.15E-03	-	-	-	1.37E-01	-	-
Trial 3	best fit	1.39E-04	9.73E-03	1.05E+04	6.93E-05	5.11E-03	2.78E-01	1.60E-02	1.36E-10	1.15E-01	5.27E-08	1.00E+06
	Lower	-	-	8.42E+03	-	4.11E-03	-	-	-	9.18E-02	-	-
Avergae		1.39E-04	9.73E-03	1.66E+04	6.93E-05	5.52E-03	2.04E-01	1.60E-02	1.36E-10	1.72E-01	5.06E-08	1.00E+06
Std. deviation		-	-	6.05E+03	-	7.86E-04	6.40E-02	-	-	5.85E-02	1.79E-09	-

Table S5. Summary of rate constants from kinetic simulation for the reaction between [**LH**Cu_3(II,II,II)(O)]$^{5+}$ and [TBA]PDACu^{II}–PhCF_3 at -60 ºC. The upper and lower limits for the rate constant were derived from the threshold of $Chi^2_{min}/ Chi^2 = 0.9$. [a] values that are dependent on k_{PT1}. [b] values that are dependent on $k_{PT2.}$ The values of k_{PT-1} and k_{PT-2} are dependent on k_{PT1} and $k_{PT2,}$ respectively, from the estimated $K_{eq}= k_{PT1}/ k_{PT-1}$ and $K_{eq}= k_{PT2}/ k_{PT-2}$ previously reported.[3,7] [c] values that are dependent on $k_{ET1.}$ [d] values that are dependent on $k_{ET2.}$ The values of k_{ET-1} and k_{ET-2} are dependent with k_{ET1} and $k_{ET2,}$ respectively, from the estimated K_{eq}, where RT $\ln K_{eq} = -nF\Delta E^o$, where, $\Delta E^o = E^o_{[\mathbf{LH}Cu3(II,II,II)(O)]5+} - E^o_{PDACuII–PhCF3}$. k_{ET3} was fixed to an effectively instantaneous rate ($k_{ET3} = 1 \times 10^6\ M^{-1}s^{-1}$). Varying this value over several orders of magnitude did not affect the simulated results.

	Rates	$k_{PT1}{}^{a}(s^{-1})$	$k_{PT-1}{}^{a}(s^{-1})$	$k_{ET1}{}^{c}(M^{-1}s^{-1})$	$k_{ET-1}{}^{c}(M^{-1}s^{-1})$	$k_{ET2}{}^{d}(M^{-1}s^{-1})$	$k_{ET-2}{}^{d}(M^{-1}s^{-1})$	$k_{PT2}{}^{b}(s^{-1})$	$k_{PT-2}{}^{b}(s^{-1})$	$k_{CPET}(M^{-1}s^{-1})$	$k_{CPET-1}(M^{-1}s^{-1})$	$k_{ET3}(M^{-1}s^{-1})$
	Upper	-	-	7.75E+04	-	2.93E-01	-	-	-	3.88E-01	-	-
Trial 1	best fit	5.00E-04	3.49E-02	3.54E+04	1.85E-04	2.63E-02	1.13E+00	3.40E-02	2.89E-10	3.48E-01	1.27E-07	1.00E+06
	Lower	-	-	6.06E+04	-	2.29E-02	-	-	-	3.03E-01	-	-
	Upper	-	-	6.14E+04	-	2.95E-02	-	-	-	5.16E-01	-	-
Trial 2	best fit	5.00E-04	3.49E-02	5.51E+04	6.93E-05	2.64E-02	1.13E+00	3.40E-02	2.89E-10	4.62E-01	4.06E-08	1.00E+06
	Lower	-	-	5.14E+04	-	2.47E-02	-	-	-	4.31E-01	-	-
	Upper	-	-	9.65E+04	-	5.95E-02	-	-	-	1.10E-01	-	-
Trial 3	best fit	5.00E-04	3.49E-02	9.27E+04	1.85E-04	5.39E-02	8.68E-01	3.40E-02	2.89E-10	1.34E-01	1.83E-07	1.00E+06
	Lower	-	-	8.95E+04	-	4.91E-02	-	-	-	1.56E-01	-	-
Avergae		5.00E-04	3.49E-02	6.11E+04	1.46E-04	3.55E-02	1.04E+00	3.40E-02	2.89E-10	3.15E-01	1.17E-07	1.00E+06
Std. deviation		-	-	2.91E+04	6.68E-05	1.59E-02	1.51E-01	-	-	1.67E-01	7.17E-08	-

Table S6. Summary of rate constants from kinetic simulation for the reaction between [**LH**Cu_3(II,II,II)(O)]$^{5+}$ and [TBA]PDACu^{II}–$PhCF_3$ at -50 ºC. The upper and lower limits for the rate constant were derived from the threshold of $Chi^2_{min}/Chi^2 = 0.9$. ^{a} values that are dependent on k_{PT1}. ^{b} values that are dependent on $k_{PT2.}$ The values of k_{PT-1} and k_{PT-2} are dependent on k_{PT1} and $k_{PT2,}$ respectively, from the estimated $K_{eq} = k_{PT1}/k_{PT-1}$ and $K_{eq} = k_{PT2}/k_{PT-2}$ previously reported.[3,7] ^{c} values that are dependent on $k_{ET1.}$ ^{d} values that are dependent on $k_{ET2.}$ The values of k_{ET-1} and k_{ET-2} are dependent with k_{ET1} and $k_{ET2,}$ respectively, from the estimated K_{eq}, where $RT \ln K_{eq} = -nF\Delta E^o$, where, $\Delta E^o = E^o_{[\mathbf{LH}Cu3(II,II,II)(O)]5+} - E^o_{PDACuII-PhCF3}$. k_{ET3} was fixed to an effectively instantaneous rate ($k_{ET3} = 1 \times 10^6\ M^{-1}s^{-1}$). Varying this value over several orders of magnitude did not affect the simulated results.

	Rates	$k_{PT1}{}^{a}(s^{-1})$	$k_{PT-1}{}^{a}(s^{-1})$	$k_{ET1}{}^{c}(M^{-1}s^{-1})$	$k_{ET-1}{}^{c}(M^{-1}s^{-1})$	$k_{ET2}{}^{d}(M^{-1}s^{-1})$	$k_{ET-2}{}^{d}(M^{-1}s^{-1})$	$k_{PT2}{}^{b}(s^{-1})$	$k_{PT-2}{}^{b}(s^{-1})$	$k_{CPET}(M^{-1}s^{-1})$	$k_{CPET-1}(M^{-1}s^{-1})$
	Upper	-	-	7.74E+00	-	4.51E-04	-	-	-	4.35E-03	-
Trial 1	best fit	2.83E-05	1.98E-03	3.96E+00	1.54E-06	2.31E-04	7.41E-01	3.30E-03	2.80E-11	2.23E-03	6.06E-08
	Lower	-	-	2.03E+00	-	1.18E-04	-	-	-	1.14E-03	-
	Upper	-	-	6.12E+00	-	3.37E-04	-	-	-	3.31E-03	-
Trial 2	best fit	2.83E-05	1.98E-03	3.13E+00	1.84E-06	1.72E-04	8.35E-01	3.30E-03	2.80E-11	1.70E-03	6.97E-08
	Lower	-	-	1.60E+00	-	8.82E-05	-	-	-	8.68E-04	-
	Upper	-	-	6.44E+00	-	5.51E-04	-	-	-	4.44E-03	-
Trial 3	best fit	2.83E-05	1.98E-03	3.30E+00	1.63E-06	2.82E-04	1.15E+00	3.30E-03	2.80E-11	2.27E-03	7.86E-08
	Lower	-	-	1.69E+00	-	1.44E-04	-	-	-	1.16E-03	-
Avergae		2.83E-05	1.98E-03	3.46E+00	1.67E-06	2.28E-04	9.09E-01	3.30E-03	2.80E-11	2.07E-03	6.96E-08
Std. deviation		-	-	4.38E-01	1.54E-07	5.50E-05	2.14E-01	-	-	3.18E-04	9.00E-09

Table S7. Summary of rate constants from kinetic simulation for the reaction between [**LH**Cu_3(II,II,II)(O)]$^{5+}$ and [TBA]PDACu^{II}–Ph$(CF_3)_2$ at -70 ºC. The upper and lower limits for rate constant were derived from the threshold of $Chi^2_{min}/Chi^2 = 0.9$. [a] values that are dependent on k_{PT1}. [b] values that are dependent on k_{PT2}. The values of k_{PT-1} and k_{PT-2} are dependent on k_{PT1} and k_{PT2} respectively, from the estimated $K_{eq} = k_{PT1}/k_{PT-1}$ and $K_{eq} = k_{PT2}/k_{PT-2}$ previously reported.[3,7] [c] values that are dependent on k_{ET1}. [d] values that are dependent on k_{ET2}. The values of k_{ET-1} and k_{ET-2} are dependent with k_{ET1} and k_{ET2}, respectively, from the estimated K_{eq}, where RT lnK_{eq} = –nFΔE^o, where, $\Delta E^o = E^o_{[\mathbf{LH}Cu3(II,II,II)(O)]5+} - E^o_{PDACuII-Ph(CF3)2}$. A subsequent k_{ET3} step is not applicable in this system, as the redox potential of [TBA]PDACu^{II}–Ph$(CF_3)_2$ is not sufficiently reducing to support an additional electron-transfer event.

	Rates	$k_{PT1}{}^{a}(s^{-1})$	$k_{PT-1}{}^{a}(s^{-1})$	$k_{ET1}{}^{c}(M^{-1}s^{-1})$	$k_{ET-1}{}^{c}(M^{-1}s^{-1})$	$k_{ET2}{}^{d}(M^{-1}s^{-1})$	$k_{ET-2}{}^{d}(M^{-1}s^{-1})$	$k_{PT2}{}^{b}(s^{-1})$	$k_{PT-2}{}^{b}(s^{-1})$	$k_{CPET}(M^{-1}s^{-1})$	$k_{CPET-1}(M^{-1}s^{-1})$
	Upper	-	-	9.70E+01	-	1.27E-03	-	-	-	3.32E-02	-
Trial 1	best fit	2.83E-05	1.98E-03	2.03E+01	1.03E-06	2.66E-04	1.11E-01	3.30E-03	2.80E-11	6.96E-03	2.47E-08
	Lower	-	-	2.18E+00	-	2.85E-05	-	-	-	7.48E-04	-
	Upper	-	-	1.59E+01	-	3.14E-04	-	-	-	3.19E-04	-
Trial 2	best fit	2.83E-05	1.98E-03	5.20E+00	4.74E-06	1.03E-04	7.73E-01	3.30E-03	2.80E-11	9.72E-04	6.20E-08
	Lower	-	-	2.13E+00	-	4.21E-05	-	-	-	3.43E-05	-
	Upper	-	-	1.21E+01	-	1.31E-03	-	-	-	1.38E-02	-
Trial 3	best fit	2.83E-05	1.98E-03	4.95E+00	2.66E-06	5.34E-04	2.37E+00	3.30E-03	2.80E-11	5.66E-03	2.13E-07
	Lower	-	-	1.62E+00	-	1.75E-04	-	-	-	1.86E-03	-
Avergae		2.83E−05	1.98E−03	1.02E+01	2.81E−06	3.01E−04	1.08E+00	3.30E−03	2.80E−11	4.53E−03	9.99E−08
Std. deviation		-	-	4.38E-01	1.54E-07	5.50E-05	2.14E-01	-	-	3.18E-04	9.00E-09

Table S8. Summary of rate constants from kinetic simulation for the reaction between [**LH**Cu_3(II,II,II)(O)]$^{5+}$ and decamethylferrocene ($Me_{10}Fc$) at -70 ºC. The upper and lower limits for the rate constant were derived from the threshold of $Chi^2_{min}/ Chi^2 = 0.9$. [a] values that are dependent on k_{PT1}. [b] values that are dependent on $k_{PT2.}$ The values of k_{PT-1} and k_{PT-2} are dependent on k_{PT1} and $k_{PT2,}$ respectively, from the estimated $K_{eq}= k_{PT1}/ k_{PT-1}$ and $K_{eq}= k_{PT2}/ k_{PT-2}$ previously reported.[3,7] [c] values that are dependent on $k_{ET1.}$ [d] values that are dependent on $k_{ET2.}$ The values of k_{ET-1} and k_{ET-2} are dependent with k_{ET1} and $k_{ET2,}$ respectively, from the estimated K_{eq}, where $RT \ln K_{eq} = -nF\Delta E^o$, where, $\Delta E^o = E^o_{[\mathbf{LH}Cu3(II,II,II)(O)]5+} - E^o_{Me10Fc}$. A subsequent k_{ET3} step is not applicable in this system, as the redox potential of $Me_{10}Fc$ is not sufficiently reducing to support an additional electron-transfer event.

Note: The rate constants summarized in Table S8 show that decamethylferrocene ($Me_{10}Fc$) and [TBA]**PDA**Cu^{II}–Ph$(CF_3)_2$ exhibit similar PCET rate constants. Given their nearly identical redox potentials (E° = −0.48 V for $Me_{10}Fc$ and −0.46 V vs Fc/Fc^+ for [TBA]PDACu^{II}–Ph$(CF_3)_2$), these results suggest that the PCET mechanisms are governed primarily by the electron transfer driving force.

	Rates	$k_1^c (M^{-1}s^{-1})$	$k_2 (s^{-1})$	$k_{-2} (s^{-1})$	$k_3 (M^{-1}s^{-1})$	$k_{-3} (M^{-1}s^{-1})$
	Upper	3.11E+02	3.87E-03	-	3.28E+04	-
Trial 1	best fit	2.66E+02	3.70E-03	3.14E-11	1.68E+04	1.38E+02
	Lower	2.37E+02	3.33E-03	-	1.08E+04	-
	Upper	2.01E+01	2.15E-03	-	7.50E+04	-
Trial 2	best fit	1.77E+01	2.03E-03	1.72E-11	2.22E+04	4.00E+01
	Lower	1.61E+01	1.92E-03	-	1.97E+03	-
	Upper	1.60E+02	5.04E-03	-	6.63E+05	-
Trial 3	best fit	8.19E+01	3.22E-03	2.74E-11	1.68E+04	1.36E+01
	Lower	3.20E+01	3.01E-03	-	1.94E+03	-

Table S9. Summary of rate constants from kinetic simulation for the reaction between [**L**Cu_3(II,II,II)(O**H**)]$^{5+}$ and $Cr(C_6H_6)_2$ at −70 ºC. The upper and lower limits for rate constant were derived from the threshold of Chi^2_{min}/ Chi^2 = 0.9.. [a] values that are dependent on k_2. The values of k_3 and k_{-3} are obtained with the known $K_{eq} = k_3/ k_{-3}$, which were obtained from prior CV simulation.[3]

References

(1) Bower, J. K.; Cypcar, A. D.; Henriquez, B.; Stieber, S. C. E.; Zhang, S. C(Sp3)-H Fluorination with a Copper(II)/(III) Redox Couple. *J. Am. Chem. Soc.* **2020**, *142* (18), 8514–8521. https://doi.org/10.1021/jacs.0c02583.

(2) Zhang, W.; Moore, C. E.; Zhang, S. Encapsulation of Tricopper Cluster in a Synthetic Cryptand Enables Facile Redox Processes from CuICuICuIto CuIICuIICuIIstates. *Chem. Sci.* **2021**, *12* (8), 2986–2992. https://doi.org/10.1039/d0sc05441k.

(3) Zhang, W.; Moore, C. E.; Zhang, S. Multiple Proton-Coupled Electron Transfers at a Tricopper Cluster: Modeling the Reductive Regeneration Process in Multicopper Oxidases. *J. Am. Chem. Soc.* **2022**, *144* (4), 1709–1717. https://doi.org/10.1021/jacs.1c10948.

(4) Hernán-Gómez, A.; Orr, S. A.; Uzelac, M.; Kennedy, A. R.; Barroso, S.; Jusseau, X.; Lemaire, S.; Farina, V.; Hevia, E. Exploiting Synergistic Effects in Organozinc Chemistry for Direct Stereoselective C-Glycosylation Reactions at Room Temperature. *Angew. Chemie - Int. Ed.* **2018**, *57* (33), 10630–10634. https://doi.org/10.1002/anie.201805758.

(5) Reese, M. S.; Bonanno, M. G.; Bower, J. K.; Moore, C. E.; Zhang, S. C-N Bond Formation at Discrete CuIII-Aryl Complexes. *J. Am. Chem. Soc.* **2023**, *145* (49), 26810–26816. https://doi.org/10.1021/jacs.3c09260.

(6) Stoll, S.; Schweiger, A. EasySpin, a Comprehensive Software Package for Spectral Simulation and Analysis in EPR. *J. Magn. Reson.* **2006**, *178* (1), 42–55. https://doi.org/10.1016/j.jmr.2005.08.013.

(7) Mondal, S.; Zhang, W.; Zhang, S. Thermodynamics of Proton-Coupled Electron Transfer at Tricopper μ-Oxo/Hydroxo/Aqua Complexes. *J. Am. Chem. Soc.* **2024**, *146* (22), 15036–15044. https://doi.org/10.1021/jacs.3c14420.

(8) Johnson, K. A.; Simpson, Z. B.; Blom, T. Global Kinetic Explorer : A New Computer Program for Dynamic Simulation and Fitting of Kinetic Data. *Anal. Biochem.* **2009**, *387* (1), 20–29. https://doi.org/10.1016/j.ab.2008.12.024.

(9) Johnson, K. A.; Simpson, Z. B.; Blom, T. FitSpace Explorer : An Algorithm to Evaluate Multidimensional Parameter Space in Fitting Kinetic Data. *Anal. Biochem.* **2009**, *387* (1), 30–41. https://doi.org/10.1016/j.ab.2008.12.025.
